# Trans-Vitisin B Targets Neuroinflammation, Oxidative Stress, and Tau Pathology to Improve Behavioral Outcomes in a Mouse Model of Parkinson’s Disease

**DOI:** 10.3390/antiox15091112

**Published:** 2026-09-03

**Authors:** Evgeny Pislyagin, Igor Manzhulo, Irina Agafonova, Anna Starinets, Ekaterina Menchinskaya, Ekaterina Chingizova, Darya Tarbeeva, Sergey Fedoreyev, Dmitry Aminin

**Affiliations:** 1 G.B. Elyakov Pacific Institute of Bioorganic Chemistry, Far Eastern Branch of the Russian Academy of Sciences, Vladivostok 690022, Russia; agafonova@piboc.dvo.ru (I.A.); ekaterinamenchinskaya@gmail.com (E.M.); martyyas@mail.ru (E.C.); tarbeeva1988@mail.ru (D.T.); fedoreev-s@mail.ru (S.F.); daminin@piboc.dvo.ru (D.A.); 2 A.V. Zhirmunsky National Scientific Center of Marine Biology, Far Eastern Branch, Russian Academy of Sciences, Vladivostok 690041, Russia; i-manzhulo@bk.ru (I.M.); anan.star13@yandex.ru (A.S.)

**Keywords:** *trans*-vitisin B, rotenone, Parkinson’s disease, neuroprotection, neuroinflammation, oxidative stress

## Abstract

Current Parkinson’s disease (PD) therapies like Levodopa (L-DOPA) only provide symptomatic relief, highlighting the need for multi-target neuroprotective agents. This study investigates the mechanisms and preclinical effects of trans-vitisin B (tVB), an oligomeric stilbene, in PD models. In LPS-stimulated HMC3 and RAW 264.7 cells, tVB (0.1–10.0 µM) significantly suppressed reactive oxygen species (ROS), nitric oxide (NO), COX-2, and pro-inflammatory cytokines (IL-1β, TNF-α), while restoring HSP70 chaperone levels to normalize proteostasis. These findings were validated in vivo using C57BL/6 mice with rotenone-induced chronic PD. Administration of tVB attenuated motor deficits (Cylinder test) and reduced pathological freezing (Open Field) and working memory impairments (Y-maze) in this model, without inducing the dyskinesia-like side effects of L-DOPA treatment in rodents. Histologically, tVB mitigated the loss of dopaminergic neurons (TH^+^) in the substantia nigra, reduced microglial activation (IBA-1^+^) and neuronal NO synthase, and suppressed pathological phosphorylated Tau protein (p-TauSer202) accumulation. Unlike L-DOPA’s direct dopaminergic stimulation, tVB’s neuroprotective efficacy is mediated through multilevel regulation of key PD pathogenetic pathways, including neuroinflammation, oxidative stress, and impaired proteostasis.

## 1. Introduction

Parkinson’s disease (PD) is a chronic neurodegenerative disorder of the central nervous system characterized by progressive loss of motor function due to the death of dopaminergic neurons in the substantia nigra of the brain, particularly within the substantia nigra pars compacta (SNpc). The degeneration of these nigrostriatal projections leads to a severe dopamine deficiency in the striatum, which underlies the classic motor symptoms of the disease. This process is closely linked to a complex set of pathological mechanisms, including chronic inflammation, oxidative stress, and cell apoptosis, which lead to neurotransmitter imbalance and the development of symptoms such as tremor, muscle rigidity, and bradykinesia. A central pathological hallmark of PD is the misfolding and aggregation of the presynaptic protein α-synuclein. These aggregated species form insoluble fibrils that constitute Lewy bodies and Lewy neurites, which propagate throughout the brain in a prion-like manner, correlating with disease progression and driving further neuroinflammation, mitochondrial dysfunction, and, ultimately, neuronal death [1]. Non-motor symptoms include cognitive decline, sleep disturbances, depression, and autonomic dysfunction, as well as dementia and memory loss. Without adequate therapy, these symptoms progress rapidly, significantly reducing both quality of life and life expectancy [2]. Current treatments are primarily aimed at managing symptoms rather than stopping or reversing the underlying process of neurodegeneration [3].

One of the key mechanisms of PD progression is neuroinflammation mediated by the activation of microglia, the primary immune cells of the central nervous system. In response to pathological stimuli such as aggregated α-synuclein, which acts as a potent danger-associated molecular pattern (DAMP), microglia become hyperactive, forming NLRP3 inflammasomes that promote the release of pro-inflammatory cytokines such as TNF-α and IL-1β [4]. While TNF-α enhances the inflammatory response and promotes neuronal degradation, cytokines such as IL-4 may play a dual role, partially modulating inflammation, although they are more often associated with disease progression. Interferons IFN-α and IFN-γ are also involved, stimulating microglia and increasing the production of nitric oxide (NO) and reactive oxygen species (ROS), which cause oxidative stress and damage to the membranes of dopaminergic neurons [5,6]. These factors not only promote cell apoptosis but also create a vicious cycle in which the accumulation of ROS and NO exacerbates inflammation, accelerating neurodegeneration and clinical deterioration [7,8]. Thus, there remains an urgent need for new therapeutic strategies that target the underlying causes of the disease.

Stilbenes are a class of polyphenolic compounds derived from plants such as grapes, where they act as protective agents against environmental stressors. Of particular interest is the oligomeric stilbene *trans*-vitisin B (tVB), a tetramer of resveratrol with a unique chemical structure consisting of phenyl rings linked by an ethylene bridge, which ensures stability and high bioavailability. Recent studies have revealed the potential therapeutic benefits of stilbene compounds derived from natural sources, such as resveratrol and pterostilbene. These polyphenolic compounds exhibit antioxidant, anti-inflammatory, and neuroprotective properties, making them promising candidates for the treatment of PD [9,10]. Oxidative stress significantly contributes to the progression of PD by damaging mitochondria and inducing apoptosis in dopaminergic neurons. Stilbenes scavenge free radicals and prevent lipid peroxidation, thereby reducing oxidative damage to neurons [9,11]. Resveratrol has been demonstrated in multiple studies to have neuroprotective, anti-inflammatory, and antioxidant characteristics and the ability to minimize amyloid-β Aβ peptide aggregation and toxicity in the hippocampus of Alzheimer’s patients, stimulating neurogenesis and inhibiting hippocampal degeneration [12].

Chronic inflammation exacerbates neurodegeneration in patients with PD. Oligomeric stilbenes suppress proinflammatory cytokines and inhibit the nuclear factor kappa B (NF-kB) signaling pathway, thereby mitigating inflammation-induced neuronal damage [9,13,14,15].

Resveratrol tetramer-tVB possesses potent antioxidant properties, effectively neutralizing ROS and preventing oxidative stress in cells. Furthermore, it exhibits pronounced anti-inflammatory effects by suppressing microglial activation and the secretion of proinflammatory cytokines, making it a potential candidate for therapeutic interventions aimed at slowing the progression of neurodegenerative diseases such as PD.

We previously demonstrated that vitisin A and *trans*-vitisin B exhibited promising neuroprotective properties by reducing mitochondrial ROS levels and cardiolipin peroxidation. This study highlighted the potential of these compounds to mitigate oxidative stress and inflammation associated with PD. It should be noted that, unlike tVB, its isomer, cis-vitisin B, did not have such properties. Furthermore, we presented new data on the antioxidant mechanisms of these stilbenes, including their ability to directly scavenge ROS [9].

Vitisin B (tVB) was shown to enhance cell viability and promote cell survival in an in vitro model using SH-SY5Y cells exposed to scopolamine-induced cytotoxicity. The therapeutic potential of vitisin B was studied in vivo using both systemic administration and direct delivery into the third ventricle of the brain in a scopolamine-induced Alzheimer’s disease AD mouse model. In both administration routes, vitisin B demonstrated a significant pro-cognitive effect, restoring multiple domains of learning and memory disrupted by scopolamine. Vitisin B was demonstrated to recover spatial working memory in the Y-maze, normalize exploratory activity in the open field, improve recognition memory in the novel object recognition (NOR) test, and enhance long-term memory retention in the passive avoidance assay. Vitisin B improved cognitive function, alleviated cholinergic deficits, increased hippocampal brain-derived neurotrophic factor (BDNF) levels, and enhanced synaptic plasticity [16].

The aim of this study is to comprehensively investigate the anti-inflammatory activity of the oligomeric stilbene *trans*-vitisin B, extracted from Alpha grape variety, and evaluate its therapeutic potential for slowing the progression of PD. Through a series of in vitro and in vivo experiments, we aim to demonstrate the mechanisms by which tVB inhibits key inflammatory and neurodegenerative processes, including reactive oxygen species and nitric oxide production, cytokine secretion, cell apoptosis, and behavioral impairments in LPS- and rotenone-induced models. Rotenone is a naturally occurring isoflavonoid derived from the roots of tropical plants, used as an insecticide, pesticide, and piscicide. It acts as a potent mitochondrial complex I inhibitor, blocking cellular respiration, and is widely utilized in research to model PD due to its neurotoxicity.

Additionally, this study focuses on analyzing the effects of tVB on Tau protein phosphorylation to quantify phosphorylated Tau (p-TauSer202) levels in substantia nigra, on the expression of the heat shock protein HSP70 as a marker of proteostasis and cellular stress, and on an immunohistochemical profile to quantify the activity of microglia (IBA-1), tyrosine hydroxylase (TH)-positive dopaminergic neurons, and neuronal NO synthase (nNOS) in the substantia nigra. This study aims to provide an evidence base for further development of the natural compound as a potential therapeutic agent against neuroinflammation in PD, including for its ability to prevent the accumulation of pathological p-Tau and restore proteostasis.

## 2. Materials and Methods

### 2.1. Plant Material

The Alpha grape variety, sampled in the southern part of the Primorsky Krai (Russia), was previously described as a source of tVB [9]; its herbarium specimen (voucher No. 267600) is deposited in the bioresource collection of the Federal Scientific Center of Agrobiotechnology of the Far East, named after A.K. Chaika (Ussuriysk, Russia).

### 2.2. Extraction and Isolation

The extraction and isolation procedure for tVB was previously described in [9]. Additionally, we used the preparative HPLC technique as the final purification step.

We carried out the preparative HPLC using a Shimadzu HPLC system equipped with an LC-20AT pump and SPD-20A detector (λ = 280 nm) (Shimadzu, Kyoto, Japan). tVB was purified using a Discovery HS-F-5-5 (Supelco Analytical, Bellefonte, PA, USA) column (5 μM, 10 mm, 250 mm). The mobile phase consisted of 1% aqueous acetic acid (A) and acetonitrile containing 1% acetic acid (B). For the analysis, the following gradient steps were programmed: 0–2 min—5–10% B, 2–4 min—10–20% B, 4–7 min—20–30% B, 7–10 min—30–40% B, 10–12 min—40–50% B, 12–17 min—50–100% B, and 17–25 min—100–5% B. The flow rate was 1 mL/min.

tVB (**6**): white, amorphous powder; UV (EtOH) λ_max_ 198, 287, 320 nm; $\alpha_{D}^{20}$ = −114°; ECD (8.03 × 10^−5^ M, CH_3_OH) λ_max_ (Δε) 204 (+80.04), 221 (−25.18), 236 (+29.42), 303 (−13.90); ^1^H NMR and 13C NMR were published in [9] in the Supplementary Materials Section (Figures S1–S11).

### 2.3. Cells Culture

Cells lines: mouse neuroblastoma Neuro-2a CCL-131™, human microglial HMC3 CRL-3304^TM^, and mouse macrophage RAW 264.7 TIB-71^TM^ were procured from the American Type Culture Collection (ATCC, Manassas, VA, USA). The cells were cultured in a 75 cm^2^ culture flask (Corning, Phoenix, AZ, USA) within a CO_2_ incubator using Dulbecco’s Modified Eagle’s Medium (DMEM) supplemented with 10% fetal bovine serum and 1% antibiotics (Biolot, St. Petersburg, Russia).

### 2.4. Analysis of ROS Levels

HMC3 and RAW 264.7 cells were seeded in black, clear-bottom 96-well plates (2.0 × 10^4^ cells/well) and pre-treated with tVB (0.1–10.0 μM) for 1 h. Inflammation was induced by adding 1.0 μg/mL LPS (*E. coli* 055:B5, Sigma Aldrich, St. Louis, MO, USA) for 24 h. ROS levels were then assessed by incubating cells with 10.0 μM DCFH-DA (Molecular Probes, Eugene, OR, USA) for 30 min at 37 °C in the dark. Fluorescence was detected at 485/520 nm (excitation/emission) using a PHERAstar FS high-speed plate reader (BMG Labtech, Ortenberg, Germany). Data analysis was performed via MARS software (v. 3.01R2), To account for potential variations in cell density, fluorescence intensity was normalized to the initial cell seeding number and subsequently expressed relative to the control group.

### 2.5. Griess Assay

To assess nitric oxide (NO) production, HMC3 cells (2.0 × 10^4^ cells/well) were treated with 1.0 μg/mL LPS and tVB for 24 h. Nitrite accumulation in the supernatant, a stable oxidation product of NO, was quantified using the Griess assay. Briefly, 100 μL of culture supernatant was mixed with an equal volume of Griess reagent (Sigma-Aldrich, St. Louis, MO, USA) and incubated for 15 min at room temperature. Absorbance was measured at 546 nm using a PHERAstar FS high-speed plate reader (BMG Labtech, Ortenberg, Germany) according to the manufacturer’s instructions.

### 2.6. ELISA Assay

HMC3 or RAW 264.7 cells (10 × 10^4^/mL) were seeded in 96- or 24-well plates and incubated for 24 h at 37 °C in a 5% CO_2_ incubator for cell attachment. After that, cells were treated with tVB at concentrations of 1.0 µM and quercetin of 10.0 µM for 1 h. Subsequently, LPS was introduced to each well at a concentration of 1.0 μg/mL, and the cells were incubated for 24 h. Cells incubated without or with LPS alone were used as negative and positive controls, respectively. The cells were gently washed with cold PBS (pH 7.2, 0.1 M) and then resuspended in fresh lysis RIPA-buffer with PMSF. The plates were then shaken (at 4 °C) for 1 h, after which the lysis buffer was collected in tubes and centrifuged. Cell lysates were immediately analyzed for TNF-α, IL-1β, and COX-2 levels using enzyme-linked immunosorbent assay kits. TNF-α (A-8756) and IL-1β (A-8766) were detected using kits (Vector-Best, Koltsovo, Russia). COX-2 was detected using the SEA699Mu kit from Cloud-Clone Corp. (Houston, TX, USA).

### 2.7. Western Blotting

HMC3 cells were seeded in Petri dishes (Ø = 60 mm) at a density of 1 × 10^5^ cells per mL in 5 mL DMEM and allowed to adhere for 24 h at 37 °C in a humidified atmosphere with 5% CO_2_. After adhesion, cells were pre-treated for 1 h with either 10.0 μM tVB or 10.0 μM quercetin. Subsequently, LPS was added to a final concentration of 1.0 μg/mL, and cells were incubated for an additional 24 h. Cells treated with vehicle only served as negative controls, while cells treated with LPS alone served as positive controls.

Following incubation, cells were harvested and lysed in RIPA buffer (Sigma-Aldrich, St. Louis, MO, USA) with PMSF. Protein concentration was measured according to the Bradford method, and equal amounts of protein were separated by electrophoresis on Precast Gel Plus Tris-Glycine 10% or 4–15% gel (WSHT, Shanghai, China). Proteins were then transferred onto a 0.45 μm PVDF membrane (Millipore Corporation, Bedford, MA, USA). The membrane was blocked with 5% bovine serum albumin (BSA) (Biolot, St. Petersburg, Russia) in Tris-buffered saline with Tween 20 (TBST) for 1 h at room temperature.

Primary antibodies were applied overnight at 4 °C: monoclonal mouse anti-HSP70 (1:1000, Invitrogen, Carlsbad, CA, USA, Cat. No. MA3-007) and monoclonal rabbit anti-glutathione synthetase (GSS) (1:500, ABclonal, Düsseldorf, Germany, Cat. No. A11557), with monoclonal mouse b-actin (1:1000, Cloud-Clone, Wuhan, China, Cat. No. MAB340Hu21) as a loading control. After washing, the membrane was incubated with HRP-Linked Caprine Anti-Mouse IgG Polyclonal Antibody (1:10000, Cloud-Clone, China, Cat. No. SAA544Mu19) and HRP-Linked Caprine Anti-Rabbit IgG Polyclonal Antibody (1:10000, Cloud-Clone, China, Cat. No. SAA544Rb19) for 1 h at room temperature. Protein bands were visualized using an enhanced chemiluminescence (ECL) detection system and imaged with a ChemiDoc MP system (Bio-Rad, Hercules, CA, USA).

Densitometric quantification of immunoreactive bands was performed using Image Lab software (Bio-Rad Laboratories, Hercules, CA, USA). The optical density of each target protein band (HSP70 and GSS) was measured and normalized to the optical density of the loading control (β-actin) obtained from the same membrane. All data were obtained from at least three independent experiments.

### 2.8. Apoptosis Analysis

After a 24 h attachment period, neuronal cells seeded at a density of 5 × 10^4^ cells per mL in a 12-well plate were treated with a 10.0 µM solution of tVB. The Neuro-2a cells were then incubated for 1 h, following which a 10.0 µM solution of rotenone was added dropwise. Incubation was continued for an additional 24 h. Thereafter, cells were detached using trypsin, washed sequentially with cold phosphate-buffered saline and 1× binding buffer, and stained with Annexin V AF 488 and propidium iodide according to the manufacturer’s protocol (Lumiprobe, Moscow, Russia). Flow cytometric analysis was performed using a NovoCyte flow cytometer (Agilent Technologies, Santa Clara, CA, USA).

### 2.9. Cell Cycle Analysis

Neuro-2a cells were seeded in 12-well plates at a density of 5 × 10^4^ cells per mL and cultured until they reached the exponential growth phase. Cells were then pre-treated with 10.0 µM tVB for 1 h, followed by the addition of 10.0 µM rotenone. Incubation continued for an additional 24 h.

At the end of the treatment period, cells were detached with trypsin, collected by centrifugation, and washed once with phosphate-buffered saline (PBS). Fixation was performed by dropwise addition of ice-cold 70% ethanol, and samples were stored overnight at 4 °C. The following day, fixed cells were treated with RNase A (100 µg/mL) for 1 h at 37 °C to remove RNA, followed by staining with propidium iodide (50 µg/mL) for 5 min in the dark at room temperature.

Cell cycle analysis was performed using a NovoCyte flow cytometer (Agilent Technologies, Santa Clara, CA, USA). The proportion of cells in G0/G1, S, and G2/M phases was quantified using appropriate software, and results are presented as mean percentages ± standard deviation.

### 2.10. Animals

Male C57Bl/6 mice (six months old, 30–32 g) purchased from the Moscow animal nursery (Stolbovaya station, Russia), the Stolbovaya Animal Nursery of the Scientific Center for Biomedical Technologies (Moscow, Russia), were used for experiments. All mice were raised in an environment with a 12 h light/dark cycle at a temperature of 22 ± 1 °C with ad libitum access to food and water. All experimental procedures with animals were carried out in accordance with the European Directive 2010/63/EC “On the protection of animals used for scientific purposes” and the rules of GOST 33044-2014 “Principles of good laboratory practice”. Work with animals was approved by the local ethics committee of the PIBOC FEB RAS, No 05/25 from 16.10.25.

### 2.11. Parkinson’s Disease Induction

After mice (*n* = 24) had adapted and were habituated to handling, they were divided into four groups randomly: (1) the control intact mice, “CTL” (*n* = 6); (2) the rotenone-treated model group, “ROT” (*n* = 6); (3): the rotenone-treated group that received trans-vitisin B at a dose of 1.0 mg/kg, “ROT + tVB” (*n* = 6); and (4) the rotenone-treated group that received Levodopa (L-DOPA) at a dose of 10 mg/kg, “ROT + LD” (*n* = 6).

Rotenone (Sigma-Aldrich, St Louis, MO, USA) was dissolved in dimethyl sulfoxide and olive oil (1:9 *v*/*v*). Dissolved rotenone was injected subcutaneously daily for 14 consecutive days at a dose of 2.5 mg/kg to construct the model. Following the 14-day injection period, a subsequent two-week post-exposure window was utilized. This timeline allows for the progression of glial activation, protein aggregation, and irreversible dopaminergic neuronal loss in the substantia nigra pars compacta, establishing a self-sustaining and progressive model of neurodegeneration [17].

#### Treatment Schedule

From day 14 after rotenone exposure, mice received:

L-DOPA (L-3,4-dihydroxyphenylalanine, USP, 12601 Twinbrook PKWY, Rockville, MD, USA), 10 mg/kg intraperitoneally (i/p), once daily for 14 days.

The dose of 1 mg/kg for tVB was selected based on the foundational preclinical pharmacokinetic study which demonstrated that doses of 0.5–1 mg/kg provide robust, reproducible, and quantifiable systemic exposure within a validated analytical range [18]. This choice also considers effective doses of structurally related stilbenes, such as resveratrol, previously evaluated in models of neurodegenerative diseases [19].

**Scheme 1:**
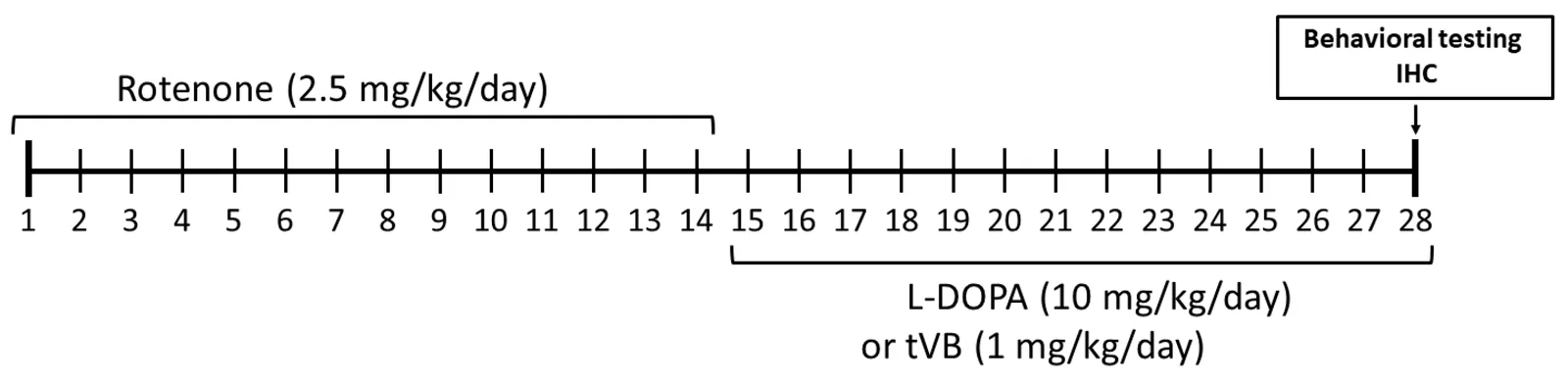
Study design and treatment schedule.

The complete timeline of rotenone, L-DOPA and tVB administration is illustrated in Scheme 1.

One day after the last injection of L-DOPA or tVB, behavioral assessments were performed. To prevent cumulative stress and minimize potential carry-over or fatigue effects, the behavioral tests were executed in a strict, predefined sequence, moving from the least to the most stressful task: Open Field → Y-maze → Cylinder test → Grip Strength. A precise 1 h resting interval was maintained between consecutive tests, during which the animals were housed in their home cages in a quiet room to ensure full behavioral and physiological recovery. No animals or data points were excluded from the analysis.

### 2.12. Behavioral Tests

All behavioral studies and subsequent data analyses were performed by an experimenter blinded to the treatment group. All animals were habituated to handling prior to the experiment. To minimize potential confounders, the order of treatments and measurements, as well as animal cage locations, was systematically randomized. All the behavioral tests were performed one day after the last injection during the light cycle between 7:00 a.m. and 7:00 p.m. The apparatus for each behavioral test was thoroughly cleaned with 70% ethanol each time before testing an animal to minimize olfactory signals. Each animal was tested 3 times with regular intervals between measurements.

#### 2.12.1. Cylinder Test Protocol

A transparent glass cylinder (height: 19.5 cm, diameter: 15 cm) was surrounded by black cardboard on three sides to minimize the effects of environmental lighting. One side of the cylinder was facing the camera for video recording. A SONY camera (Sony Corporation, Tokyo, Japan, Carl Zeiss, Virio Tessar, 70×) was used during experiments. Each mouse was individually placed into the cylinder and video-recorded for 3 min. During the testing, external noise and light fluctuations were avoided to ensure that these factors did not influence the behavior of the mice. The cylinder was then washed with water and dried with 70% ethanol before another mouse was placed in it. The changes in the behavioral reactions of mice were assessed against the background of the development of the rotenone model. The number of mouse climbs was calculated. A climb is when the mouse climbs onto its hind limbs, raises both forelimbs above shoulder level, and lands. Lifting of one front limb was not taken into account.

#### 2.12.2. Open Field Test Protocol

To assess locomotor activity and anxiety-like behavior, an Open Field test (OFT) was performed using a square, grey opaque plastic box (40 × 40 × 40 cm). To eliminate olfactory cues, the arena was thoroughly washed with 70% ethanol and allowed to dry completely between different pairs of mice. Testing was conducted during the light phase of the circadian cycle, under controlled ambient conditions (dim lighting at 30 lux, low background noise). Animals were transported in their home cages to the behavioral testing room to habituate to the room environment for at least 60 min prior to any procedure. Throughout the study, mice were group-housed (4–6 per cage), and the pairs tested in the OFT were always established cagemates from the same home cage to prevent aggression or novelty-induced social interactions. One day prior to the formal test, each pair underwent an arena habituation session, where they were placed in the empty OFT arena together and allowed to freely explore for 5 min. Data from this habituation session were not included in the final analysis. Our study utilized an unconventional approach of testing two mice simultaneously. This experimental setup was specifically chosen as a refinement strategy to enhance translational validity and minimize confounding factors associated with our PD model. In this model, neurodegeneration and neuroinflammation induce high baseline anxiety. Placing a singly housed mouse into a novel, brightly lit arena acts as a potent anxiogenic stimulus, often triggering a profound freezing response or high thigmotaxis. Such fear-driven behaviors mask the actual motor capacity of the animal, confounding the interpretation of locomotor activity. The presence of a familiar cagemate serves as a social buffer, significantly mitigating novelty-induced anxiety and stress. This encourages spontaneous exploratory and locomotor behavior, providing a more accurate, less anxiety-confounded readout of pure motor function [20]. Crucially, this protocol was applied uniformly across all experimental groups; thus, the social context remained a controlled, constant variable across all conditions. On the test day, the two cagemates were gently placed simultaneously in the center of the open field, and their behavior was recorded for 5 min using a SONY digital camera (Carl Zeiss, Vario-Tessar, 70×) mounted directly above the arena. Animal tracking and behavioral metrics were analyzed using the automated software “ToxTrac” (v.2.84, Umeå University, Linnéusväg, SE-901 87, Sweden). To address potential pseudoreplication and ensure data independence, each mouse was uniquely marked, allowing the software to track individual trajectories. The individual values within each pair were subsequently averaged to yield a single mean value per pair, which served as the statistical unit (*n* = 1) per pair for analysis. The evaluated parameters included average speed (cm/min), total distance traveled (mm), total freezing time (sec), and the number of freezing events.

#### 2.12.3. Y-Maze Test Protocol

The Y-maze test is based on the principle of spontaneous alternation behavior (SAB). SAB describes the natural tendency of rodents to choose a different arm of the maze than the one they previously visited. Each choice is statistically dependent on the preceding ones, reflecting the animal’s working memory and exploratory drive. The Y-maze test was conducted 1 h after the Open Field test. Prior to testing, an initial 5 min habituation session was performed, during which the mice were allowed to freely explore the maze. For the experiment, each mouse was placed in the center of the labyrinth and video was recorded for 5 min using Bandicam software (version 7.1.4.2458, Bandicam Company, Seoul, Republic of Korea).

The experimental setup consisted of three identical arms oriented at 120° to each other (length: 40 cm, width: 7 cm, height: 25 cm). At the start of the session, the mouse was placed in a designated “start” arm. The analysis includes recording the sequence of entries into the arms to evaluate spontaneous alternation. An entry was recorded only when the animal entered an arm with all four paws. Similarly, an exit was defined by all four paws leaving the arm [21].

#### 2.12.4. Grip Strength Test Protocol

Muscle strength was assessed using a BIO GS3 device (Bioseb, Vitrolles, France) equipped with a grip plate on day 28 of the experiment. Testing was performed by the same trained experimenter in the morning (8:00 a.m. to 10:00 a.m.) to minimize within-group variability. Testing all animals within this narrow 2 h window and strictly counterbalancing the treatment groups across this timeframe ensured that any circadian variation affected all experimental groups equally.

The mouse was carefully placed on the grip plate, allowing it to grasp it with its forelimbs and hindlimbs (a total limb strength test). After the mouse established its grip, the experimenter grabbed it by the base of its tail and gently pulled it backward in a straight line parallel to the surface of the device until the grip weakened. The device automatically recorded the maximum force in grams (g).

Three consecutive trials were performed for each mouse, after which the average value for each group of mice in the experiment was calculated and used for analysis.

### 2.13. Immunohistochemistry

Experimental animals were sacrificed 28 days after the onset of the PD model. The same cohorts of mice were used for both behavioral testing and subsequent molecular and histological analyses to ensure consistency. All surgical procedures were performed under deep anesthesia with 3.5% isoflurane (Laboratories Karizoo, SA, Barcelona, Spain) in 100% oxygen (VetFlo, Kent Scientific Corporation, Torrington, CT, USA). Following behavioral assays, animals from each experimental group were randomly allocated to specific downstream immunohistochemistry analyses according to the statistical power requirements of each method. Mice were transcardially perfused with PBS (4 °C), 0.1 M, pH 7.2, then with 10% formalin prepared in PBS (4 °C), 0.1 M, pH 7.2. The brains were then removed and fixed for 24 h in fresh 10% formalin. The brains were then washed in PBS, dehydrated, embedded in paraffin, and sectioned at a thickness of 10 μm (in the sagittal projection) on a Leica RM 2245 rotary microtome (Leica, Wetzlar, Germany). For immunohistochemical studies, brain sections were deparaffinized and incubated in 3% H_2_O_2_ to block endogenous peroxidase activity. Sections were then washed in 0.1 M PBS (pH 7.2), and nonspecific antibody binding was blocked by incubation in 2% bovine serum albumin (Sigma-Aldrich, Darmstadt, Germany) with 0.01% Triton X-100 (Sigma-Aldrich, Darmstadt, Germany) for 60 min. Next, the sections were incubated with primary antibodies (Iba-1 (1:2000, ab178846, Abcam, Cambridge, UK), nNOS (1:1000, ab40662, Abcam, Cambridge, UK), TH (1:200, T 489, Vector Laboratories, Newark, NJ, USA)) and Phospho-Tau (Ser202) (1:200, Affinity Biosciences, Changzhou, China) for 24 h at 4 °C. Negative controls (samples without treatment with primary antibodies) were used in the study. One day after washing, the sections were incubated in a solution of secondary antibodies (ab178846, Abcam, Cambridge, UK) for 15 min. After washing, sections were incubated for 10 min with streptavidin (ab64269, Abcam, Cambridge, UK). Staining was performed using the DAB chromogen (Abcam, Cambridge, UK) for 5 min. Sections were then counterstained with hematoxylin for 10 min. After rinsing with running water, sections were dehydrated and mounted in mounting medium (CS705, Dako, Denver, CO, USA). Images of the substantia nigra were acquired using a Zeiss Axio Imager microscope, an AxioCam 503 camera, and AxioVision 4.7.1 software (Zeiss, Münster, Germany).

For image processing and analysis, ImageJ 1.48 software (NIH, Bethesda, MD, USA) was used. For the analysis, at least 50 images of the substantia nigra were used for each animal group. The number of TH- and nNOS-positive neurons was counted in every 7th brain section using the formula: d = (10^9^ × *n*)/(S × 10), where d is the cell density; 10^9^ is the conversion factor from μm^2^ to mm^3^; n is the number of immunopositive cells; S is the area of the substantia nigra (μm^2^); and 10 is the section thickness (μm). The area of immunopositive staining for TH-positive nerve fibers, pTAU202-positive tangles and Iba-1-positive microglia was measured in every 7th brain section using the IHC Toolbox plugin for ImageJ software (NIH, Bethesda, MD, USA). All measurements were performed by an operator blinded to the identity of the sections.

### 2.14. Statistical Analysis

All data obtained throughout the study were analyzed using appropriate statistical methods. Graph plotting and statistical evaluations were performed using GraphPad Prism 4.00 software (GraphPad Software, San Diego, CA, USA) or SigmaPlot 14.0 software (Systat Software Inc., San Jose, CA, USA). Data are presented as mean ± SEM (standard error of the mean).

The normality of the data distribution within each experimental group was rigorously evaluated using the Shapiro–Wilk test. For all datasets that satisfied the assumption of a normal distribution (*p* > 0.05 in the Shapiro–Wilk test), statistical significance was determined using parametric one-way analysis of variance (ANOVA) followed by Tukey’s post hoc test for multiple comparisons. Values of *p* < 0.05, *p* < 0.01, and *p* < 0.001 were considered statistically significant. Detailed information regarding the specific statistical methodology applied to each dataset is explicitly provided in the respective figure legends.

## 3. Results

### 3.1. tVB Inhibits LPS-Induced ROS and NO Production in HMC3 and RAW 264.7 Cells

**Figure 1:**
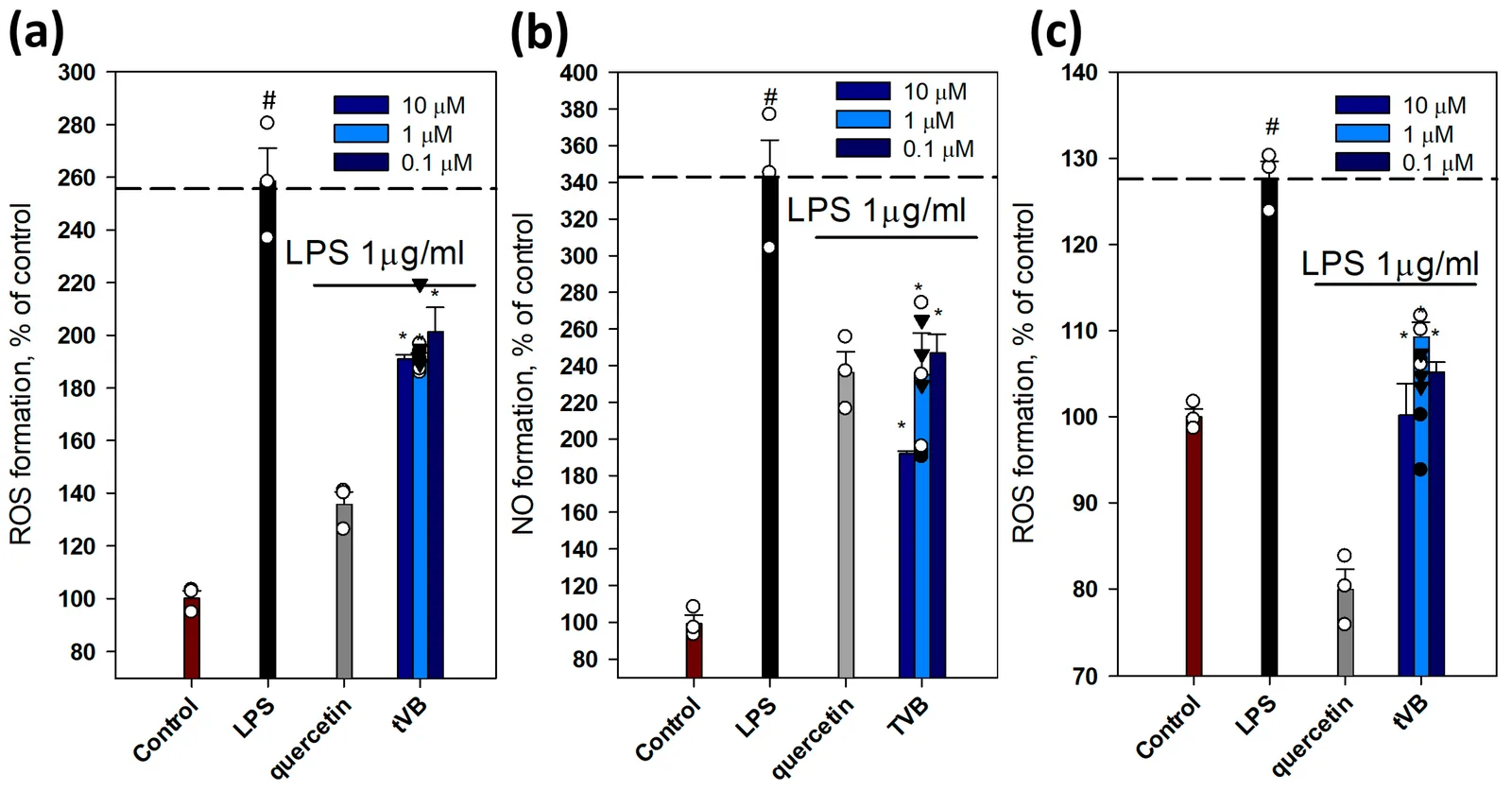
Effect of *trans*-vitisin B (tVB) (10.0, 1.0 and 0.1 μM) and quercetin 10.0 μM on reactive oxygen species (ROS) levels in HMC3 (**a**) and RAW 264.7 cells (**c**), and NO level in HMC3 (**b**) in cells treated with LPS 1.0 μg/mL. Each bar represents the mean ± SEM of the mean from three independent replicates. (*) indicates *p* < 0.05 compared to lipopolysaccharide (LPS)-treated cells (*n* = 3). (#) indicates *p* < 0.05 for negative control compared to positive control (LPS) (one-way ANOVA, post hoc Tukey test). The dotted line indicates the positive control level. The symbols (open circles, filled circles, and triangles) represent individual data points. Differences between control and LPS-treated cells were considered statistically significant.

Previously, we demonstrated that the studied substance is able to inhibit the production of ROS in Neuro-2a cells under the action of 6-OHDA and rotenone [9]. In the present study, the ability of compound tVB to inhibit ROS production in HMC3 microglia cells under the action of LPS (1 mg/mL) was studied (Figure 1a,b). One-way ANOVA revealed a significant overall treatment effect on ROS production in HMC3 cells (*F*(5, 12) = 21.569, *p* < 0.001). Subsequent post hoc analysis confirmed that exposure to LPS resulted in a significant increase in ROS production by 155.6 ± 8.1% compared to the untreated control group (*p* < 0.001), confirming the successful establishment of the in vitro inflammatory model.

The inhibitory effect of the test compound was compared with the effect of the standard antioxidant quercetin at a concentration of 10.0 mM, which significantly reduced the intracellular amount of ROS by 46.3 ± 1.4% compared to the LPS-treated positive control group (*p* < 0.001). Pre-treatment of cells with tVB at concentrations of 10.0, 1.0, and 0.1 mM demonstrated a statistically significant, dose-dependent decrease in ROS levels by 25.3 ± 1.1% (*p* < 0.01), 25.7 ± 1.3% (*p* < 0.05), and 21.3 ± 3.6% (*p* < 0.05), respectively, compared to the LPS-treated positive control group (Figure 1a). For nitric oxide production, the overall one-way ANOVA also demonstrated a significant effect across groups (*F*(5, 12) = 6.932 (*p* < 0.01)). Tukey’s post hoc investigation of the Griess analysis data revealed a significant reduction in nitrite formation compared to the level in LPS-activated cells. Specifically, nitric oxide levels were significantly reduced by 28.3 ± 2.9% (*p* < 0.05), 31.7 ± 11.3% (*p* < 0.05), and 44.2 ± 0.5% (*p* < 0.01) at 0.1, 1.0, and 10.0 mM tVB, respectively, relative to the LPS-treated group (Figure 1b). Furthermore, the protective efficacy of tVB was evaluated in mouse RAW 264.7 macrophages. One-way ANOVA indicated a highly significant variance among the treatment groups (*F*(5, 12) = 26.519 (*p* < 0.001)). Tukey’s post hoc test verified that tVB at concentrations of 10.0, 1.0, and 0.1 mM significantly lowered ROS accumulation by 21.5 ± 5.1% (*p* < 0.01), 14.4 ± 1.3% (*p* < 0.05), and 17.6 ± 1.5% (*p* < 0.05), respectively, compared to the corresponding LPS-treated positive control group (Figure 1c).

### 3.2. Evaluation of the Anti-Inflammatory Activity of tVB in the Presence of LPS

The effect of tVB on the production of the cytokines IL-1β and TNF-α in HMC3 microglia was assessed using the ELISA method. Additionally, the effect of tVB on the activity of murine cyclooxygenase-2 (COX-2) in RAW 264.7 cells stimulated with LPS was studied. Glutathione synthetase (GSS) levels were determined by Western blotting.

**Figure 2:**
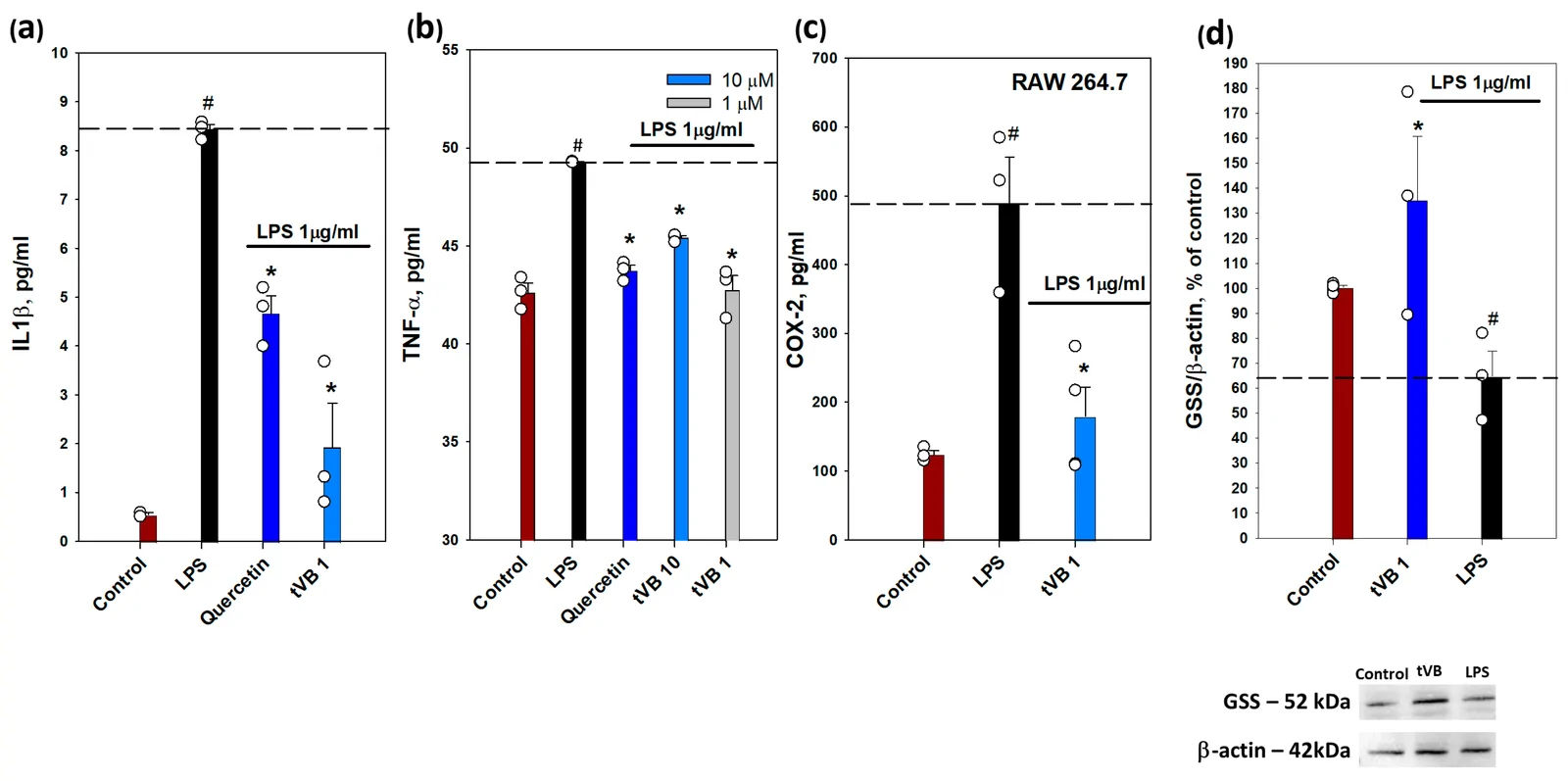
Effect of *trans*-vitisin B (tVB) on the levels of IL-1β (**a**), TNF-α (**b**), and GSS (**d**) in HMC3 cells and COX-2 (**c**) in RAW 264.7 cells treated with LPS (1.0 μg/mL). The levels of IL-1β (**a**), TNF-α (**b**), and COX-2 (**c**) were determined by ELISA. GSS levels (**d**) were determined by Western blotting. Data are presented as mean ± SEM (*n* = 3). The dashed horizontal line indicates the level of the positive control group (cells treated with LPS alone) (*) indicates *p* < 0.05 compared to cells treated with the inducer (LPS) alone, (#) indicates *p* < 0.05 for negative control compared to positive control (LPS) (one-way ANOVA, post hoc Tukey test). Circles represent individual biological replicates.

For IL-1β secretion, one-way ANOVA indicated a highly significant variance among the treatment groups (*F*(3, 8) = 32.080 (*p* < 0.001)). Subsequent post hoc analysis confirmed that LPS predictably caused a sharp increase in the production of the pro-inflammatory cytokine IL-1β in microglia compared to the untreated control group (*p* < 0.001), confirming the successful induction of the inflammatory process. Incubation of HMC3 cells with tVB (1.0 mM) in the presence of LPS led to a significant suppression of IL-1β secretion by 77.1 ± 10.4% (*p* < 0.05) compared to the LPS-treated positive control group (Figure 2a).

Regarding TNF-a secretion (Figure 2b), the overall one-way ANOVA demonstrated a significant effect across groups (*F*(4, 10) = 80.251 (*p* < 0.001)). Tukey’s post hoc investigation revealed that LPS exposure significantly elevated TNF-a levels compared to the untreated control group (*p* < 0.001). In turn, tVB demonstrated a concentration-dependent inhibitory effect; it significantly suppressed TNF-a levels by 11.4 ± 1.5% at a concentration of 1.0 mM (*p* < 0.001) and by 7.9 ± 0.3% at a higher concentration of 10.0 mM (*p* < 0.05), relative to the LPS-treated group.

Furthermore, the overall analysis of the pro-inflammatory enzyme COX-2 activity showed a significant difference among groups (*F*(2, 6) = 20.074 (*p* < 0.01)). Post hoc testing verified that tVB at a concentration of 1.0 mM helps to reduce the level of COX-2 by 61.3 ± 6.9% (*p* < 0.01) relative to the LPS-treated group (Figure 2c).

Finally, one-way ANOVA for GSS protein expression confirmed a significant overall treatment effect (*F*(2, 6) = 9.413 (*p* < 0.05)). Western blot analysis on HMC3 microglial cells demonstrated that LPS treatment significantly reduced GSS expression by 39% compared to the untreated control group (*p* < 0.05). Conversely, co-treatment with tVB counteracted this effect, significantly increasing GSS levels by 108.2% compared to the LPS-treated group (*p* < 0.05; Figure 2d).

Given that pro-inflammatory mediators released by activated macrophages/microglia are known to trigger neuronal apoptosis and disrupt cell cycle progression, we next evaluated the effects of tVB on apoptosis and cell cycle regulation in neuronal cells.

### 3.3. Effects of tVB on Apoptosis and Cell Cycle Regulation

**Figure 3:**
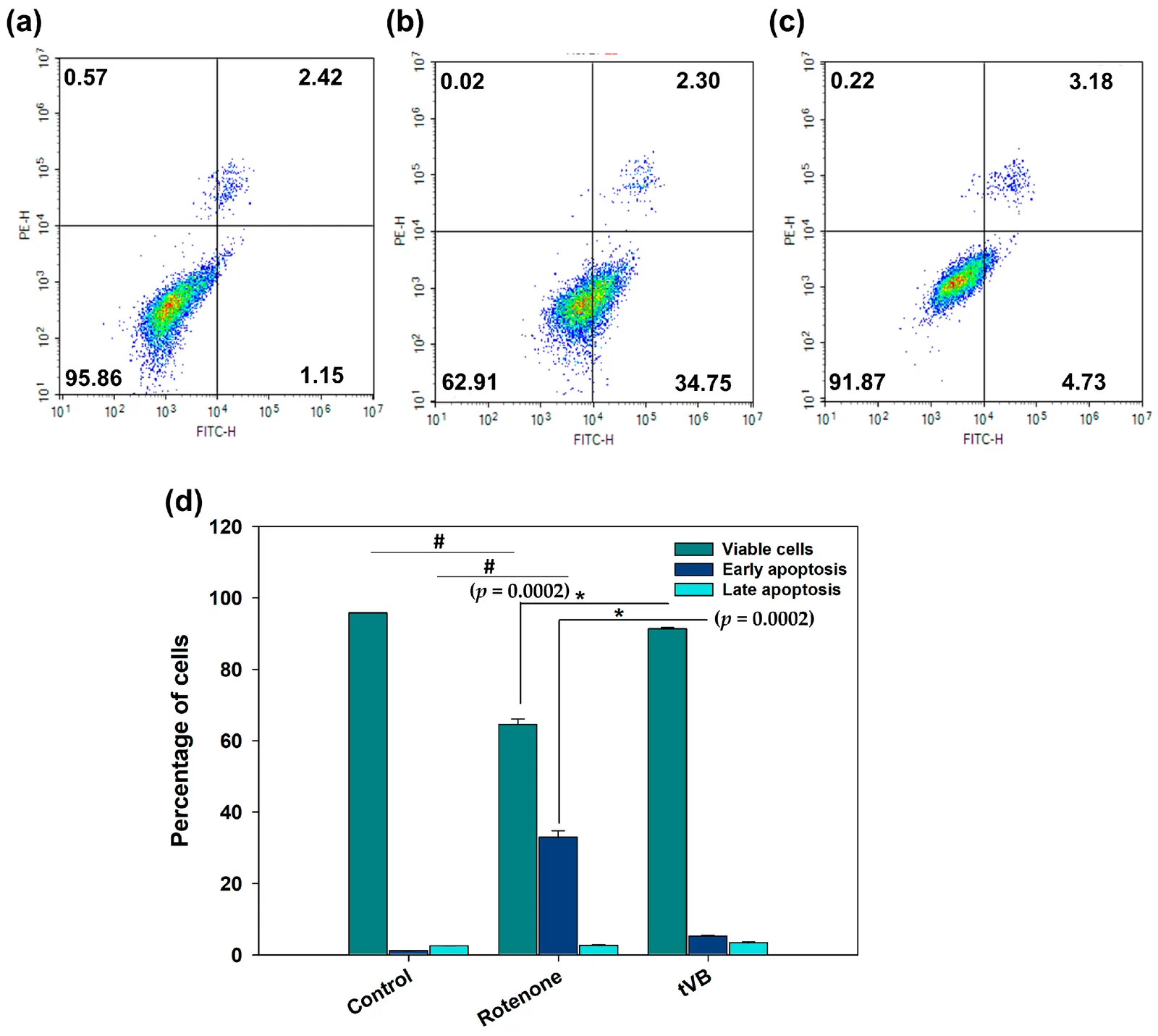
The effect of *trans*-vitisin B (tVB) on apoptosis profile in rotenone-treated Neuro-2a. Flow cytometry assay for Annexin V-AF 488/PI staining: control (**a**), rotenone 10 μM (**b**) and tVB 10 μM (**c**). Quantitative calculation of the data obtained by flow cytometry (**d**). Data are presented as means ± SEM (*n* = 3). (*) indicates a statistically significant difference compared to the untreated control group (*p* < 0.05); (#) indicates a statistically significant difference compared to the rotenone-treated positive control group (*p* < 0.05) (one-way ANOVA, post hoc Tukey test).

We analyzed the effect of tVB on the apoptosis profile in rotenone-treated Neuro-2a cells by flow cytometry using fluorescent annexin V conjugate as a fluorescent dye. One-way ANOVA revealed a significant overall treatment effect on the percentage of apoptotic and viable cells (*F*(8, 18) = 25.524 (*p* < 0.001)). Subsequent Tukey’s post hoc analysis determined significant changes in the apoptosis profile of Neuro-2a cells following 24 h rotenone exposure. Specifically, the percentage of early apoptotic cells increased after rotenone treatment from 1.15% to 34.75% compared to the untreated control group (*p* < 0.001), confirming the successful induction of apoptotic cell death in our disease model (Figure 3). Notably, pre-treatment of cells with 10.0 mM of tVB effectively counteracted the neurotoxic effect of rotenone. Post hoc comparisons verified a significant decrease in the proportion of living cells in the rotenone-only group (*p* < 0.001), whereas the number of viable cells in the tVB-treated group was significantly preserved and remained comparable to the untreated control (*p* < 0.01; Figure 3).

**Figure 4:**
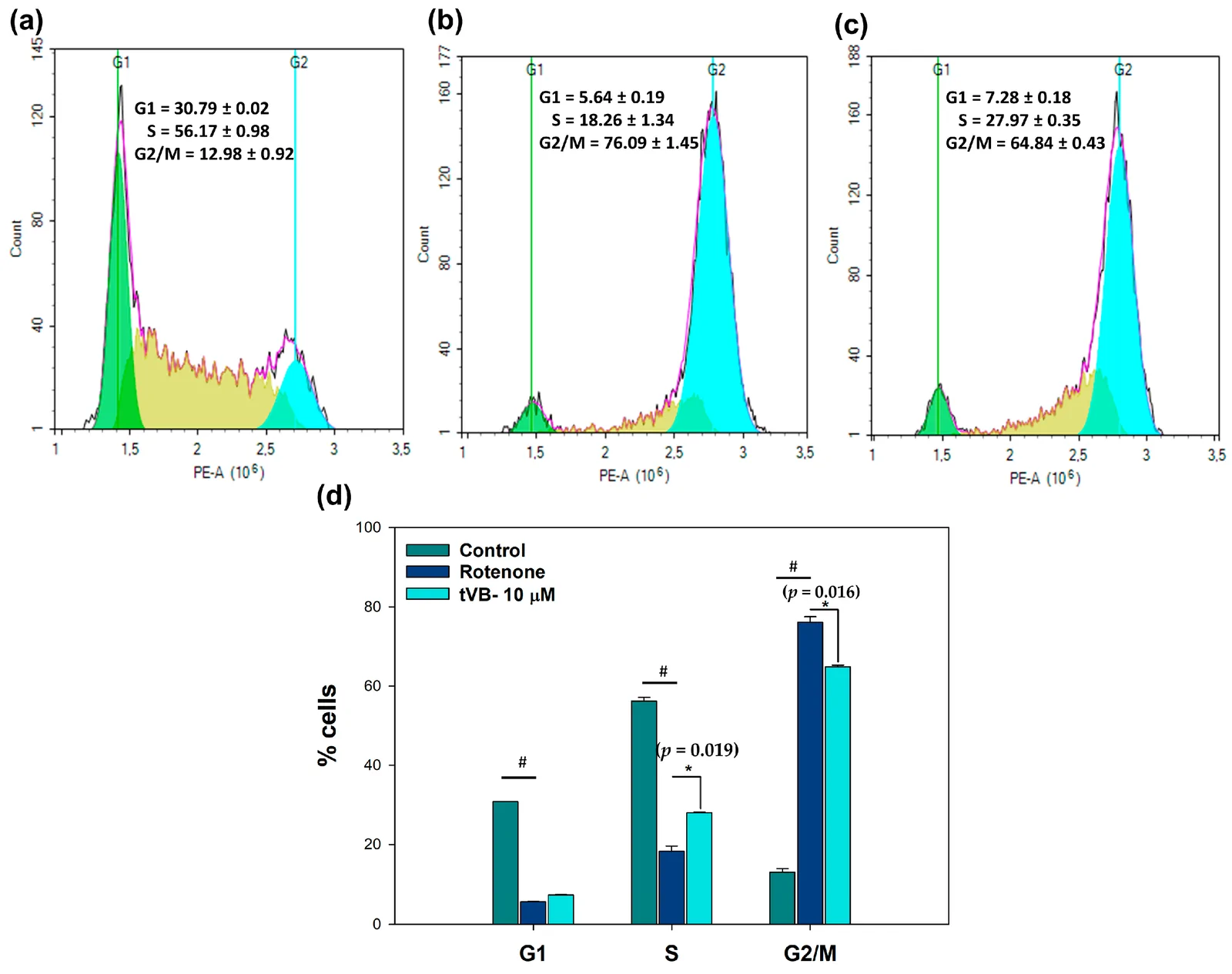
Effect of *trans*-vitisin B (tVB) on the Neuro-2a cell cycle altered by rotenone: control (**a**), rotenone 10 μM (**b**) and tVB 10.0 μM (**c**). The cell cycle was analyzed using fluorescent flow cytometry. The figure shows the percentage of cells in each stage of the cell cycle (**d**). Data are presented as means ± SEM (*n* = 3). (*) indicates a statistically significant difference compared to the untreated control group (*p* < 0.05); (#) indicates a statistically significant difference compared to the rotenone-treated positive control group (*p* < 0.05) (one-way ANOVA, post hoc Tukey test).

Additionally, we determined the effect of tVB on cell cycle restoration in neuronal cells in the presence of rotenone. The overall one-way ANOVA demonstrated highly significant differences across the treatment groups regarding cell cycle phase distribution (*F*(8, 18) = 72.961 (*p* < 0.001)). Post hoc analysis indicated that rotenone successfully arrested the cell cycle in the G2/M phase (*p* < 0.001), while significantly reducing the number of cells in the G1 and S phases (*p* < 0.05). Pre-incubating cells with tVB for 1 h promoted cell cycle normalization in cells treated with the neurotoxin. Specifically, while the percentage of rotenone-treated cells trapped in the G2/M phase was 76.1 ± 1.45%, pre-treatment with tVB significantly reduced this population to 64.8 ± 0.4% relative to the rotenone-treated positive control group (*p* < 0.05; Figure 4).

### 3.4. In Vivo Experiments

#### 3.4.1. Assessment of Cognitive Impairment in a Mouse Model of PD Using tVB

**Figure 5:**
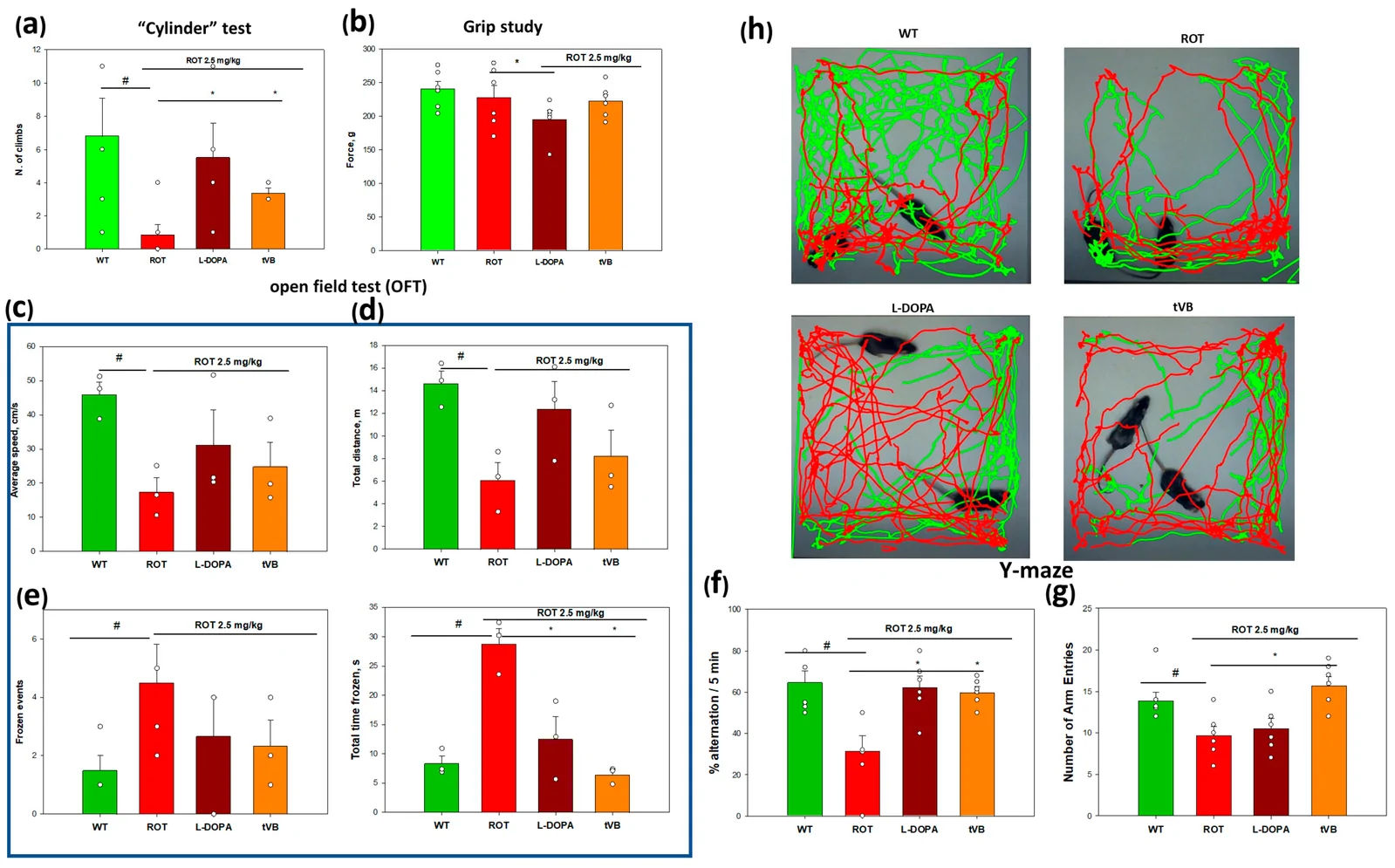
The effect of *trans*-vitisin B (tVB) on the spontaneous locomotor activity of mice with rotenone (ROT)-induced PD. The effect of tVB on the number of rearings in the Cylinder test (**a**); on the grip strength of mice (**b**); and on the average speed of mice (**c**), total distance traveled (**d**), total time spent in a frozen state (**e**), and the number of freezing episodes in the Open Field test (**e**) after ROT administration. Representative locomotion tracks of mice in the Open Field test (**h**). Spontaneous alternation frequency (**f**) and number of arm entries (**g**). Data are presented as mean ± SEM (*n* = 6 per group; for the Open Field test, the data points represent the average of 2 mice). (*) indicates *p* < 0.05, compared to the group of mice treated with rotenone only, (#) indicates a statistically significant difference compared to the rotenone-treated positive control group (*p* < 0.05) (one-way ANOVA, post hoc Tukey test).

The neurotoxin rotenone was used as an inducer of a PD model. For this purpose, C57BL/6 mice were administered rotenone subcutaneously at a dose of 2.5 mg/kg once daily for 14 days, and then disease development was allowed for an additional 14 days according to the protocol [17]. The compound tVB at a dose of 1.0 mg/kg or L-DOPA at a dose of 10.0 mg/kg was administered intraperitoneally 24 h after the last rotenone injection, daily for 14 days. Efficacy was assessed 14 days after the cessation of rotenone administration, and within 24 h after the last tVB administration, using the Cylinder test, “Open Field test”, Y-maze, and Grip Strength test (Figure 5).

#### 3.4.2. Cylinder Test

In the Cylinder test, which is designed to assess asymmetric motor activity and spontaneous forepaw rearing in mice, one-way ANOVA revealed a significant overall treatment effect on vertical exploratory behavior (*F*(3, 20) = 14.884, *p* < 0.001). Subsequent Tukey’s post hoc analysis demonstrated a significant decrease in motor activity in the group treated with rotenone; compared to the control group, rotenone-treated mice showed a 63.2% reduction in the number of spontaneous rearings (*p* < 0.05), indicative of the pronounced motor dysfunction characteristic of Parkinsonism models.

Administration of L-DOPA led to a significant recovery of motor function, with the number of forepaw rearings increasing 5.6-fold (*p* < 0.01) compared to the rotenone group. This indicates a functional activation of the dopaminergic system and confirms the validity of the model. Similarly, treatment with tVB also resulted in a statistically significant improvement in motor activity, where the number of rearings increased 3.0-fold compared to the rotenone group (*p* < 0.01), although it did not reach the level of the Levodopa effect. This points to the potential neuroprotective or modulating activity of tVB, which can partially compensate for the rotenone-induced dopaminergic degradation (Figure 5a). In summary, the Cylinder test demonstrated that rotenone treatment led to a significant decrease in contralateral forelimb use, which was significantly attenuated by both L-DOPA and tVB administration, with L-DOPA showing a greater magnitude of effect.

#### 3.4.3. Grip Strength Test

To assess the motor strength of the forelimbs, the Grip Strength test was used with the BIO-GS3 apparatus (Bioseb, Vitrolles, France). In contrast to the Cylinder test, where significant motor dysfunction was observed after rotenone induction, the average grip strength in the rotenone group was not statistically different from the control group. This indicates the preservation of basic muscular strength despite the presence of dopaminergic degradation. This finding is consistent with literature data confirming that rotenone primarily disrupts the neural circuits responsible for coordination and movement initiation, rather than peripheral muscular strength [22].

However, in the group treated with L-DOPA, a significant reduction in grip strength (14% (*p* < 0.05)) was recorded compared to both the control and rotenone groups. In contrast, treatment with tVB did not lead to a decrease in grip strength—the values remained at the control level and were significantly higher than those in the L-DOPA group (Figure 5b).

This contrast between the effects of the two therapies suggests that the decrease in grip strength with Levodopa may be associated with the development of dyskinesia—involuntary, hyperkinetic movements that often occur with chronic L-DOPA use and can impair the mouse’s ability to hold and consistently grip the apparatus bar. In contrast, tVB did not induce such side effects, while maintaining a therapeutic effect in the Cylinder test, highlighting its potential advantage as a more selective and safer neuroprotective agent. Thus, the data from the Grip Strength test demonstrate that L-DOPA, despite its effectiveness in restoring spontaneous motor activity, can induce functional impairments related to dyskinesia, whereas tVB exhibits a more favorable safety profile, preserving motor strength while improving coordination.

#### 3.4.4. Open Field Test

In the chronic rotenone exposure model, a significant decrease in motor activity was observed. This manifested in a reliable reduction in average movement speed and total distance traveled, as well as a significant increase in both the total freezing time and the frequency of freezing episodes (Figure 5c–e,h). These changes correspond to the hypokinesia and increased anxiety-like behavior characteristic of neurodegenerative models of Parkinsonism.

Treatment with L-DOPA (10.0 mg/kg) and tVB (1.0 mg/kg) did not lead to a significant restoration of average speed or distance traveled compared to the rotenone group. However, both compounds showed a trend towards partial improvement in motor activity.

The most significant effect was the restoration of the “total freezing time” parameter. One-way ANOVA revealed a significant overall difference among the experimental groups for this measure (*F*(3, 20) = 16.973, *p* < 0.05). Subsequent post hoc analysis demonstrated that tVB (1.0 mg/kg), similar to L-DOPA (10.0 mg/kg), significantly reduced the duration of freezing compared to the rotenone group (*p* < 0.05). Notably, tVB reduced it to the level of the control group (*p* > 0.05), thereby completely normalizing this behavioral measure.

Thus, both tVB and L-DOPA demonstrate a significant effect on behavioral markers of anxiety and/or motor inhibition in the rotenone-induced model of Parkinsonism.

#### 3.4.5. Y-Maze Test

In the chronic rotenone-induced PD model, a significant and persistent impairment of spatial working memory was observed. This manifested in a reliable decrease in the spontaneous alternation rate on day 28 after rotenone injection compared to the control group (Figure 5f). Spontaneous alternation, a reliable indicator of prefrontal cortex and hippocampal integrity, dropped from 64.5% in the control group to 31.2% on day 28 (*p* < 0.05 vs. control group), indicating progressive cognitive dysfunction, characteristic of the stages accompanied by degeneration of dopaminergic and cholinergic pathways. In parallel, a significant decrease in the total number of arm entries was noted on day 28 compared to the control group (*p* < 0.05), pointing to hypokinesia and reduced general locomotor activity, rather than solely a cognitive deficit (Figure 5g).

Treatment with tVB (1.0 mg/kg) on day 28 led to a significant increase in the total number of arm entries by 90.7% compared to the rotenone group (*p* < 0.05), while L-DOPA (10.0 mg/kg) caused a statistically significant restoration of motor activity by 98.9% compared to the rotenone group (*p* < 0.05). Both drugs significantly normalized the cognitive parameter compared to the rotenone group (*p* < 0.05), despite the differences in their effects on motor activity. This indicates that restoration of spatial working memory does not require complete recovery of motor function and may be due to the neuroprotective, antioxidant, or modulating effects of tVB on limbic and cortical structures, and, for L-DOPA, on dopaminergically mediated improvement in cognitive flexibility.

### 3.5. Immunohistochemistry Analysis

#### 3.5.1. tVB Reduces the Loss of Dopaminergic Neurons in the SNpc

**Figure 6:**
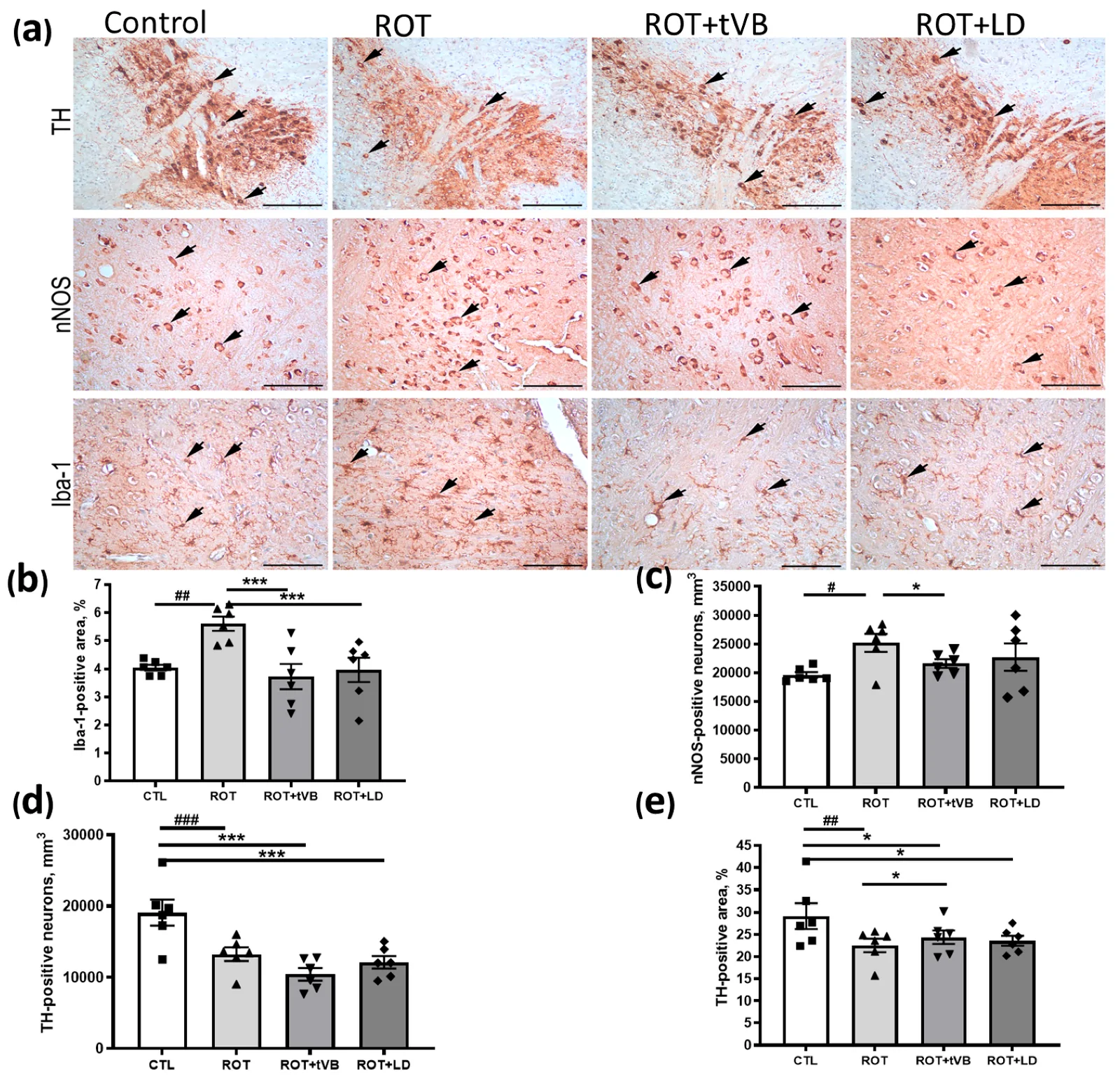
Distribution of TH-positive, nNOS-positive neurons, and Iba-1-positive microglia (immunopositive cells are indicated by arrows) in the substantia nigra of the brain in a PD model following therapy with stilbene and L-DOPA. Sections were counterstained with hematoxylin. Scale bar: TH—200 μm, nNOS and Iba-1—100 μm (**a**). The number of TH-positive neurons (**d**), nerve fibers (**e**), nNOS-positive neurons (**c**), and Iba-1-positive microglia (**b**) in the substantia nigra of the brain. Data are presented as mean ± SEM, *n* = 6 (number of animals per group). (*) indicates *p* < 0.05, (***) indicates *p* < 0.001, (#) indicates *p* < 0.05, (##) indicates *p* < 0.01, (###) indicates *p* < 0.001, indicating a statistically significant difference compared to the rotenone-treated positive control group (one-way ANOVA, post hoc Tukey test). The geometric symbols represent individual data points.

To assess the effect of tVB on neurotoxicity induced by rotenone administration, an analysis of the number of tyrosine hydroxylase (TH)-positive dopaminergic neurons in the substantia nigra pars compacta (SNpc) was performed. As shown in Figure 6a, the rotenone group exhibited significant decreases in TH-positive staining area (of 31.1%) and the number of TH-positive neurons (of 36.5%) compared to the untreated baseline control group, confirming the successful establishment of the PD model. One-way ANOVA revealed a significant overall difference among the experimental groups in the TH-positive staining area (*F*(3, 20) = 7.501, *p* < 0.01). Subsequent post hoc analysis demonstrated that administration of tVB at a dose of 1.0 mg/kg led to a substantial restoration of the TH-positive staining area, increasing it by 22.0 ± 0.8% compared to the rotenone group (*p* < 0.05) (Figure 6d,e). The immunohistochemistry data confirm that treatment with tVB at a dose of 1.0 mg/kg effectively prevents the loss of TH-positive neurons in the substantia nigra induced by the neurotoxin. However, in the L-DOPA (LD) group, there was no significant increase in TH-positive staining area compared to the rotenone group (*p* > 0.05).

#### 3.5.2. tVB Suppresses Rotenone-Induced Glial Cell Activation in the SNpc

Neuroinflammation, characterized by excessive activation of glial cells, plays an important role in the degeneration and death of dopaminergic neurons in the SNpc in PD [23]. As shown in Figure 6b, exposure to Rotenone induced strong gliosis in the SNpc. IBA-1 is a well-known marker of microglia [24]. The number of IBA-1-immunoreactive cells significantly increased under the influence of Rotenone by 39.2% compared to the untreated control group (*p* < 0.01). One-way ANOVA revealed a significant overall difference among the experimental groups in the number of IBA-1-positive cells (*F*(3, 20) = 15.584, *p* < 0.001). Subsequent post hoc analysis demonstrated that tVB attenuated the IBA-1-positive microglial response induced by Rotenone treatment by 30.7 ± 5.3% relative to the rotenone-treated positive control group (*p* < 0.001).

Neuroinflammation is also characterized by an increase in the level of the nNOS marker in brain cells of the SNpc in PD [25]. As shown in Figure 6c, exposure to Rotenone caused an increase in nNOS levels in the SNpc. The level of nNOS significantly increased under the influence of Rotenone compared to the untreated control group by 35.8 (*p* < 0.01). One-way ANOVA showed a significant overall effect of the treatment on nNOS levels (*F*(3, 20) = 6.320, *p* < 0.05). Subsequent post hoc comparisons revealed that tVB attenuated the nNOS response induced by Rotenone treatment by 17.7 ± 2.9% (*p* < 0.05). At the same time, L-DOPA did not show a significant reduction in nNOS levels in the SNpc compared to the rotenone group (*p* > 0.05). Taken together, these results demonstrate that tVB attenuated microglial activation and protected neurons from Rotenone-induced damage.

#### 3.5.3. tVB Suppresses Rotenone-Induced p-TauSer202 Formation in SNpc

**Figure 7:**
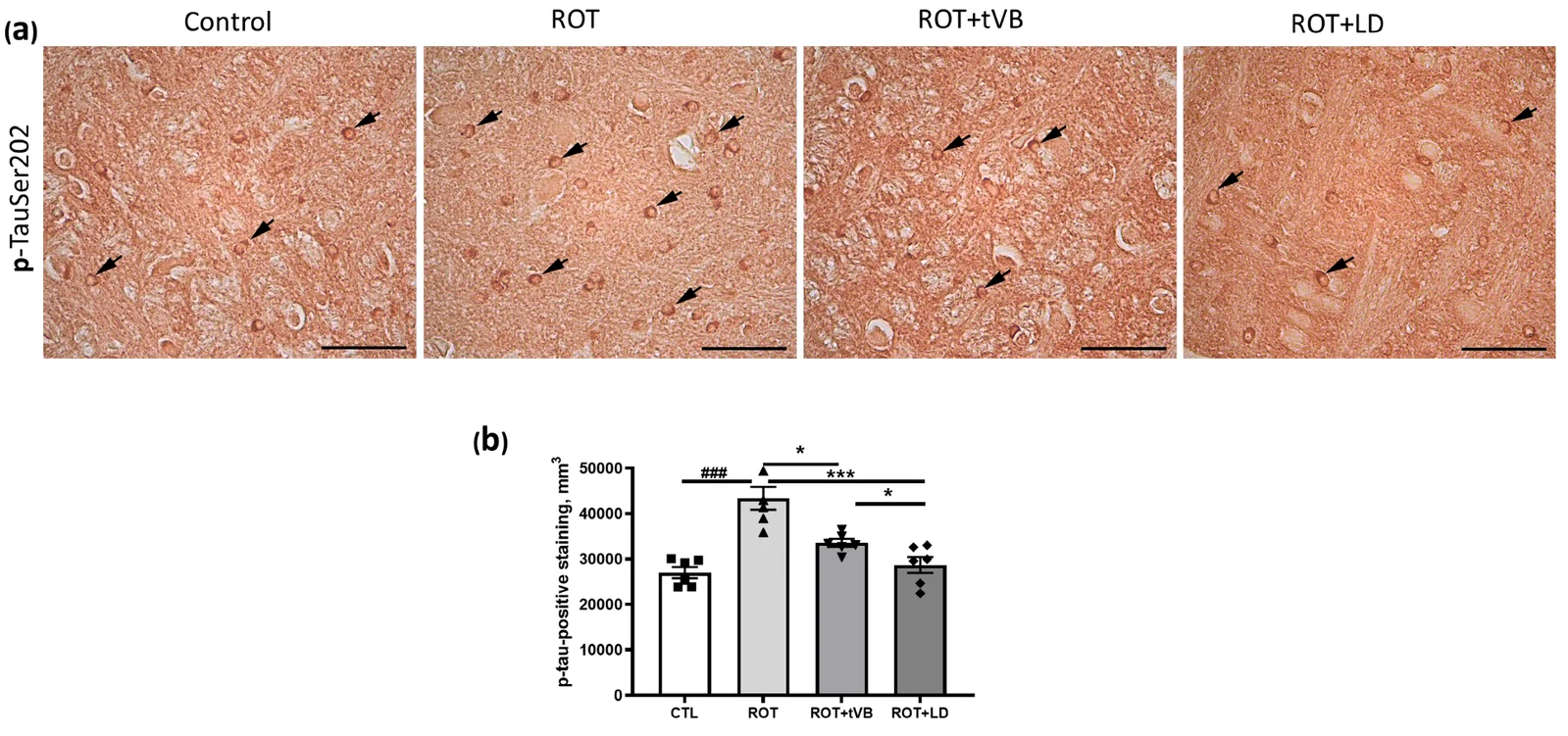
Immunohistochemical staining of p-TauSer202 in SNpc brain sections of PD model mice (**a**). Neurofibrillary tangles (ROT arrows) and dystrophic neurites were visible in the substantia nigra, observed in concentrated areas. The number of Tau-positive tangles in the substantia nigra of the brain (**b**). Data are presented as mean ± SEM, *n* = 6 (number of animals per group). * *p* < 0.05, *** *p* < 0.001, (###) indicates *p* < 0.001, indicating a statistically significant difference compared to the rotenone-treated positive control group (one-way ANOVA, post hoc Tukey test). The geometric symbols represent individual data points.

p-TauSer202 stains pathological tau protein structures in SNpc brain sections in rotenone-induced PD models. To determine whether the p-TauSer202 antibody recognizes the pathology in SNpc brain sections in models of PD, we performed immunohistochemical analysis on SNpc sections taken from *n* = 6 males (in each group) in a model of PD. Intense staining of the tau protein was observed in the Rotenone group. Neurofibrillary tangles were observed in SNpc brain sections (Figure 7a). At higher magnification, the SNpc subfield showed intense staining of neurofibrillary tangles. In SNpc sections in the rotenone group, there was a 58.3% increase in such tangles compared to the untreated baseline control group. Taken together, these results show that tau protein pathologies were stained with the p-TauSer202 antibody in the PD model. One-way ANOVA revealed a significant overall difference among the experimental groups in the number of neurofibrillary tangles (*F*(3, 20) = 17.39, *p* < 0.001). Subsequent post hoc analysis demonstrated that in the group treated with tVB and Levodopa, such tangles were observed in a much smaller amount in SNpc sections, showing a reduction of 23.9 ± 3.1% (*p* < 0.001) and 35.0% (*p* < 0.001), respectively, compared to the rotenone group (Figure 7b).

#### 3.5.4. tVB Enhances HSP70 Expression in HMC3 Microglial Cells Under LPS-Induced Inflammatory Stress

To evaluate the neuroprotective potential of the compound tVB, we investigated its effect on the expression of heat shock protein HSP70, a key molecular chaperone that plays a central role in maintaining proteostasis and ensuring cell survival under stress conditions. LPS, which induces microglial activation and pronounced oxidative stress, was used as a model of neuroinflammation associated with the degeneration of dopaminergic neurons.

**Figure 8:**
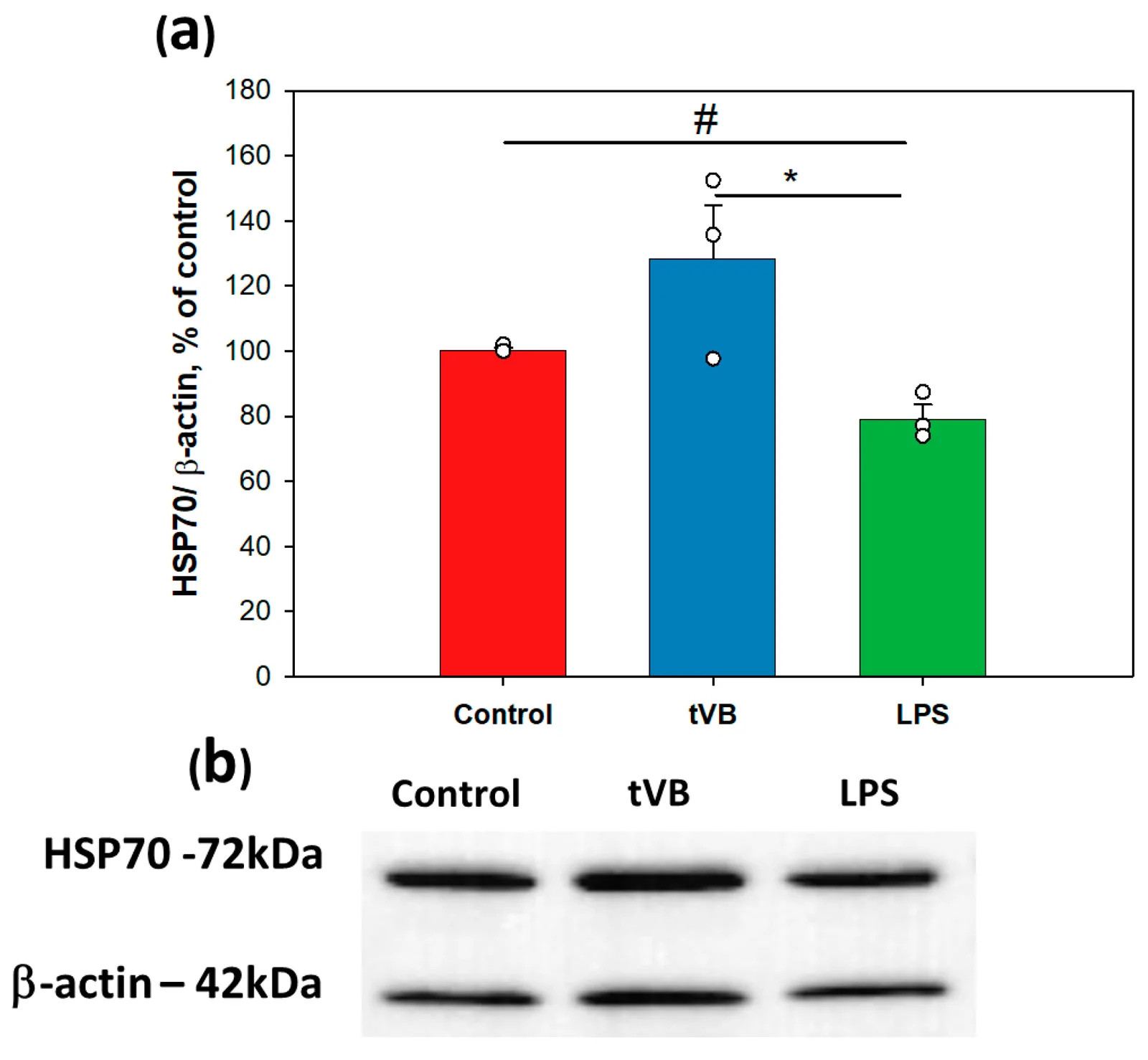
Effect of tVB on HSP70 levels in HMC3 cells. (**a**) Representative immunoblots. (**b**) Densitometric analysis of the HSP70/β–actin ratio. Data are presented as mean ± SEM, *n* = 3. (*) indicates *p* < 0.05 compared to the LPS group, (#) indicates *p* < 0.05 for negative control compared to the positive control (LPS) (one-way ANOVA, post hoc Tukey test).

Cultures of HMC3 microglial cells were treated with LPS (1.0 μg/mL, 24 h) in the presence or absence of tVB (10.0 μM). HSP70 expression levels were assessed by Western blotting. The results showed that in the control group, HMC3 cells demonstrated moderate constitutive expression of HSP70, characteristic of the physiological state of microglia. Treatment with LPS led to a significant decrease in HSP70 levels compared to the untreated baseline control group (by 20.5%, *p* < 0.05), indicating an impairment of cellular defense mechanisms and a depletion in the adaptive response to inflammatory stress. One-way ANOVA revealed a significant overall difference among the experimental groups for HSP70 expression levels (*F*(3, 20) = 8.630, *p* < 0.01). Subsequent post hoc analysis demonstrated that pre-treatment of cells with tVB (10.0 μM) significantly restored HSP70 expression (by 61.8% compared to the LPS group, *p* < 0.05) (Figure 8). This may indicate that tVB activates or enhances cellular chaperone defense mechanisms, compensating for the LPS-induced suppression of HSP70.

## 4. Discussion

In the context of our previous studies, where tVB and vitisin A were identified as the most promising neuroprotective stilbenoids, the present work extends these findings by focusing not only on efficacy but also on the mechanisms of protection of neuronal cells. This differs from the previous study, which emphasized the comparative activity of eight compounds across four toxicity models. Specifically, tVB demonstrated a unique ability for early (within 1 h) induction of SOD following exposure to rotenone and 6-OHDA, which correlated with maximal suppression of mitochondrial ROS and prevention of cardiolipin peroxidation (a marker of early mitochondrial dysfunction). This effect was not replicated by any of the other compounds, including its stereoisomer cis-vitisin B, highlighting the critical role of spatial configuration in the biological activity of stilbenes [9].

However, the fact that tVB retained its efficacy across all four models, including the MPP^+^ model, which is the most relevant for the sporadic form of PD, makes it the only candidate capable of acting on several key pathogenetic nodes: oxidative stress, mitochondrial dysfunction, inflammation, and the impairment of the antioxidant response. This multifaceted action, however, is not limited to neurons; a key component of it is the modulation of microglial activity, the main driver of neuroinflammation in the progression of neurodegenerative diseases [26].

Microglia play an important role in maintaining brain homeostasis but become dysregulated during the pathogenesis of PD. Certain oligomeric stilbenes regulate the microglial phenotype towards an anti-inflammatory state [27], promoting neuroprotection instead of neurotoxicity.

In our study, we investigated the oligomeric stilbene tVB, which has previously demonstrated a neuroprotective effect in a model of PD [9] for its ability to exert an anti-inflammatory effect on HMC3 and RAW 264.7 cells under the influence of LPS. Given that peripheral immune activation by macrophages plays a pivotal role in driving and exacerbating central neuroinflammation during PD progression, evaluating both systemic and resident brain immune cells provides a comprehensive view of tVB’s therapeutic potential.

The current findings indicate that tVB exerts a potent anti-inflammatory effect by downregulating ROS and NO production. Furthermore, tVB treatment effectively reduces the expression of IL-1β and TNF-α in HMC3 microglia and COX-2 in RAW 264.7 macrophages. Notably, similar to tVB, resveratrol (RSV) inhibits IL-1β production and TNF-α-induced signaling, further reinforcing these anti-inflammatory effects in cellular models mimicking neuroinflammation during PD pathogenesis [28]. These findings offer a novel interpretation of the neuroprotective potential of this compound: its effects are mediated not only through direct action on neurons but also via profound regulation of neuro–immune interactions within the central nervous system (CNS).

The suppression of IL-1β and TNF-α represents a central mechanism of neuroprotection. These cytokines activate microglia and astrocytes, promoting chronic inflammation, oxidative stress, and apoptosis of dopaminergic neurons in the substantia nigra [29]. Our observation of reduced cytokine levels following tVB treatment aligns with data showing that inhibition of these molecules enhances neuronal survival and is associated with attenuated motor deficits in PD models, such as those induced by MPTP and rotenone. The suppression of IL-1β is particularly significant in the context of PD, as this cytokine is linked to the activation of the NLRP3 inflammasome, which has recently been shown to play a key role in neurodegeneration in PD. Our findings suggest that tVB may interfere with this cascade, potentially explaining its anti-inflammatory effects.

In addition to its immunomodulatory action, tVB exhibits pronounced antioxidant properties, significantly reducing ROS and NO levels in LPS-stimulated HMC3 microglial cells. Experiments using fluorescent probes (DCFH-DA for ROS and Griess reagent for NO) demonstrated that pre-treatment with tVB (0.1–10.0 µM) reduces ROS production by 21–25% and NO levels by 28–44% compared to the LPS-induced group. This suppression correlates with increased activity of key antioxidant enzymes such as superoxide dismutase (SOD) [9] and glutathione synthetase (GSS), thereby suggesting activation of the Nrf2-dependent antioxidant response. Since excess NO reacts with superoxide to form peroxynitrite, which causes protein nitration and DNA damage. The reduction in its synthesis through NO inhibition represents a critical element of neuroprotection [30]. These results suggest that tVB exerts dual neuroprotective effects by neutralizing oxidative damage and suppressing the inflammatory cascade, both of which are hallmark features of PD pathology.

This positions tVB as a potential candidate for further investigation in the context of both inflammation and oxidative damage. The reduced expression of COX-2, an enzyme responsible for the synthesis of pro-inflammatory prostaglandins (particularly PGE_2_), further confirms the ability of tVB to interrupt inflammatory signaling at the level of eicosanoid pathways. PGE_2_, in turn, enhances glial cell activation and disrupts synaptic homeostasis, leading to neurotoxicity. Therefore, COX-2 inhibition represents an important additional pathway through which tVB may protect neurons from inflammation-mediated damage [31].

This study extends previous in vitro data by confirming the neuroprotective potential of tVB in an in vivo model of PD induced by rotenone [9]. The present investigation represents the first comprehensive in vivo validation of the neuroprotective behavior-modifying potential of tVB in a chronic rotenone-induced Parkinsonism model in C57BL/6 mice. The obtained data not only confirm the substantial preclinical efficacy of tVB in attenuating motor and cognitive impairments in this model but also reveal its unique action profile, which fundamentally differs from classical L-DOPA therapy. A key finding is that tVB appears to act on multiple mechanisms underlying the model’s pathology rather than simply masking symptoms, leading to improvement in both motor and cognitive outcomes without the dyskinesia-like effects associated with L-DOPA in this model.

An attenuation of rotenone-induced motor deficits was observed, with tVB acting as a compensator rather than a dopamine substitute. In the Cylinder test, tVB demonstrated a 3-fold increase in the number of spontaneous forepaw wall touches compared to the rotenone-only (ROT) group, indicating significant improvement in asymmetric motor function. Although the effect of tVB was less pronounced than that of L-DOPA (5.6-fold recovery), it remained statistically significant and, importantly, was not accompanied by reduced muscle strength. Specifically, a 14% reduction in grip strength was observed in the L-DOPA group, whereas tVB treatment preserved baseline muscle strength. While the exact mechanism behind this L-DOPA-induced decline requires further investigation, it may reflect confounding motor impairments or abnormal hyperkinetic movements often seen with pulsatile dopaminergic stimulation. The absence of such effects with tVB in this model indicates a lack of excessive D1 dopamine receptor activation, highlighting it as an interesting compound for further long-term preclinical assessment.

Thus, tVB demonstrates a “neuroprotective” approach to motor symptomatology: it does not replace dopamine but rather appears to preserve the functional integrity of dopaminergic neurons, improving their capacity for natural signal transmission. This reinforces our prior reported data, establishing a link between tVB’s ability to induce SOD and protect cardiolipin with a broader role in suppressing the oxidative and inflammatory drivers of neuronal loss in the substantia nigra.

In the Open Field test, rotenone induced pronounced hypokinesia (reduced speed and distance) and hyperfreezing, which are classic manifestations of anxiety and motor inhibition in this PD model. Although neither tVB nor L-DOPA restored overall activity to control levels, both compounds completely normalized freezing duration, with tVB achieving full restoration of this parameter to control levels.

This effect is particularly significant because hyperfreezing represents one of the least understood yet most disabling aspects of PD, often resistant to L-DOPA therapy and associated with cognitive and emotional impairments. The ability of tVB to normalize this parameter without inducing hyperkinesia underscores its differentiated action on neural networks rather than merely providing systemic dopaminergic stimulation.

The most noteworthy finding was the complete restoration of spontaneous alternation in the Y-maze in both treatment groups, tVB and L-DOPA, despite the differences in their effects on motor activity. While L-DOPA is known to improve cognitive flexibility through activation of D1/D2 dopamine receptors in the prefrontal cortex [32], tVB normalized this cognitive measure in the absence of complete motor recovery (average speed and distance traveled remained reduced). This provides preclinical evidence that tVB acts on mechanisms that are at least partially independent of direct dopaminergic stimulation. A 28% reduction in the number of arm entries in ROT mice indicated general hypokinesia, but tVB increased this number by 90.7%, while L-DOPA increased it by 98.9% (not significant). This suggests that tVB not only improves cognitive flexibility, but also stimulates general motor initiative.

Upon completion of the in vivo experiments, histological analysis was performed. Histological examination of the substantia nigra pars compacta in C57BL/6 mice subjected to chronic rotenone exposure not only confirmed the successful establishment of the PD model but also demonstrated for the first time the multifaceted neuroprotective effects of tVB, including preservation of dopaminergic neurons, suppression of pathological protein aggregates, and modulation of glial activation. These findings, complemented by in vitro results from HMC3 microglial cells, form a comprehensive picture: tVB acts not as a symptomatic treatment but as a regulator of multiple cellular resilience pathways targeted by the model’s pathogenetic mechanisms.

Immunohistochemical staining for tyrosine hydroxylase (TH) (a marker of dopaminergic neurons) showed pronounced degeneration in the ROT group: a 36.5% decrease in the number of TH^+^ neurons and a 31.1% decrease in TH-positive staining area compared to controls. These data are fully consistent with the literature on the rotenone-induced PD model and confirm the progressive loss of dopaminergic neurons in the SNpc [17,33]. In the group receiving tVB (1.0 mg/kg, intraperitoneally), the number of TH-positive neurons increased by 22.0% compared to ROT, and the TH-positive staining area increased by 24.3%. This indicates a direct neuroprotective effect of tVB, preserving the viability of dopaminergic neurons, rather than simply stimulating their activity. At the same time, despite improved behavioral parameters, L-DOPA did not significantly increase the number of TH^+^ neurons (vs. ROT). L-DOPA masks neuron loss by enhancing TH synthesis in the remaining cells. tVB preserves neurons, attenuating their loss and allowing natural TH expression to recover by preserving cell mass.

One possible mechanism of tVB’s neuroprotective action is the regulation of the cell cycle and suppression of apoptosis in dopaminergic neurons induced by rotenone. As our data showed, rotenone causes a pronounced arrest of the cell cycle in the G2/M phase, where the percentage of cells in this phase increased to 76% compared to the control, while a significant decrease in the proportion of cells in the G1 and S phases was observed, indicating impaired proliferative activity and the onset of stress-induced cyclostasis. Such arrest in G2/M is a characteristic sign of cellular stress associated with mitochondrial damage and accumulation of DNA damage, and often precedes the initiation of caspase-dependent apoptosis in neurons in PD models.

Pre-incubation with 10.0 μM tVB for 1 h normalized the cell cycle profile: the proportion of cells in the G2/M phase decreased to 64%, which corresponds to a restoration of the balance between the G1 and S phases. This indicates that tVB not only blocks the toxic effect of rotenone, but also actively restores the functional integrity of the cell cycle, allowing neurons to overcome the stress barrier and avoid irreversible arrest. It is worth noting that evaluating cell cycle dynamics in the proliferating Neuro-2a clonal cell line is a well-established and widely accepted method in the literature. This model is actively utilized to study cell cycle regulation, differentiation, and toxicity in response to various natural compounds, including monomeric and oligomeric stilbenes. Therefore, the assessment of the N2a cell cycle allows for a comprehensive evaluation of how the oligomeric stilbene tVB affects cellular division and checkpoint progression under experimental stress conditions [34].

Neuroinflammation, mediated by microglial activation and neuronal expression of neuronal NO synthase (nNOS), is a key driver of PD progression. ROT caused a 35.8% increase in nNOS^+^ neurons, indicating increased oxidative stress and nitrosative damage. tVB reduced nNOS expression by 17.7%, while L-DOPA had no significant effect.

ROT also caused a 39.2% increase in the number of IBA-1^+^ microglial cells, which corresponds to the transition of microglia to an activated, inflammatory state (amoeboid morphology). tVB reduces this activation by 30.7%, returning the morphology of microglia to that observed in the control group. L-DOPA had no significant effect, confirming its inability to modulate neuroinflammation and explaining its limited long-term effectiveness in this model. This confirms that tVB suppresses not only the microglial but also the neuronal component of neuroinflammation by reducing the level of toxic NO, which inhibits the mitochondrial respiratory chain and promotes apoptosis.

Pathological phosphorylation of Tau protein (p-TauSer202), previously considered exclusively a marker of Alzheimer’s disease, is increasingly found in the SNpc in PD and correlates with the progression of cognitive impairment [35,36].

Immunohistochemistry for p-TauSer202 in the SNpc showed intense staining of neurofibrillary tangles (NFTs) in the ROT group. The number of such structures increased by 58.3% compared to the control. tVB significantly reduced the number of p-Tau^+^ tangles by 23.9%, while L-DOPA reduced them by 35.0%. This indicates that tVB not only protects dopaminergic neurons but also prevents the formation of pathological Tau protein aggregates, which are considered one of the most therapy-resistant signs of neurodegeneration.

Our findings indicate that the regulatory effect of tVB on p-Tau is indirect and multi-targeted. By suppressing the release of pro-inflammatory cytokines (IL-1β and TNF-α) and reducing ROS and NO production in activated microglia, tVB attenuates the upstream kinase activation cascade known to trigger aberrant Tau hyperphosphorylation [37,38]. Furthermore, the observed upregulation of the molecular chaperone HSP70 by tVB provides an additional mechanism that facilitates the refolding and clearance of hyperphosphorylated Tau species, thereby maintaining structural proteostasis within the nigrostriatal system.

Tau protein pathology in the SNpc is a new biomarker of PD progression associated with cognitive decline. The ability of tVB to suppress p-TauSer202 provides the first preclinical evidence of its effect on proteostasis in this PD model, suggesting it may be a promising candidate for further investigation in the context of PD with cognitive impairment.

Several preclinical animal models demonstrate the efficacy of oligomeric stilbenes against PD-like phenotypes induced by toxins such as rotenone, MPTP, or 6-hydroxydopamine. For example, treatment with oligomeric stilbenes reduced α-synuclein aggregates, improved locomotor deficits, and prevented dopaminergic neuron loss in rodent models [39,40,41].

The heat shock protein 70 (HSP70) plays a critical protective role in the pathogenesis of PD by acting as a molecular chaperone. Its main function is to prevent the accumulation and aggregation of pathological proteins, in particular a-synuclein and pTau, the main component of Lewy bodies [42,43].

A 34% reduction in HSP70 expression during inflammatory stress induced by LPS indicates a primary disruption of proteostasis; the key chaperone that controls protein aggregation and suppresses “danger” signals is suppressed [43,44,45]. The 62% restoration of HSP70 levels in LPS-treated HMC3 microglial cells under the action of tVB indicates a fundamentally new mechanism of neuroprotection for this compound. Preliminary administration of tVB compensates for this deficiency, which probably allows microglia to: maintain the ability to refold damaged proteins, limit the formation of toxic aggregates, including phosphorylated tau, and reduce the production of pro-inflammatory cytokines.

Thus, the enhancement of HSP70-mediated chaperone protection is now considered not only as a cytoprotective effect, but also as an “anti-aggregation” mechanism that prevents the formation of p-Tau^+^ structures previously described in the SNpc in PD. The pharmacological scope of tVB is notably broad in these preclinical models: it protects neurons while suppressing tau phosphorylation and restoring healthy microglial activity. This integrative profile warrants further preclinical evaluation of tVB as a potential approach for targeting the dual progression of motor and cognitive deficits associated with PD.

**Limitations:** Despite its promising findings, the present study has several limitations that necessitate a cautious interpretation of the data. First, a prominent limitation is that trans-vitisin B (tVB) was evaluated at a single dose level of 1.0 mg/kg. While this specific dose was selected based on established pharmacokinetic feasibility [18] and effective doses of structurally related stilbenes, evaluating only one dose level of tVB limits the scope of our pharmacological conclusions. Specifically, the present data do not allow for the determination of a dose–response relationship, the minimum effective dose, or the optimal therapeutic dose of tVB. Furthermore, the potential therapeutic window, as well as alternative efficacy or safety profiles that might emerge at different doses of tVB, remains uncharacterized. Second, the rotenone-induced model of Parkinsonism does not fully replicate the complex progressive neurodegeneration characteristics of sporadic PD in humans. Third, all experiments were performed in C57BL/6 mice; studies of the effects on genetically modified models (e.g., α-synuclein transgenic) and on animals with age-related senescence, which more adequately mimic human pathology, are needed.

Fourth, the present study provides only indirect pharmacological evidence suggesting the potential involvement of specific cytoprotective signaling pathways (e.g., Nrf2, NF-κB, MAPK). Because direct molecular validation—such as Western blotting for phosphorylated pathway components, nuclear translocation assays, siRNA-mediated knockdown, or reporter gene analyses—was not performed, these findings demonstrate statistical associations rather than direct causal relationships. Consequently, the data should be viewed as phenomenological rather than strictly mechanistic, and the proposed pathway engagement remains hypothetical. Dedicated experiments are planned to dissect the precise signaling cascades mediating tVB’s effects.

Fifth, another methodological limitation is that all four behavioral assays were conducted sequentially within a single day. Although we strictly controlled the testing order, progressing from the least aversive to the most physically demanding task (Open Field → Y-maze → Cylinder test → Grip Strength), and instituted a 1 h resting period between trials to mitigate stress, the potential for subtle carry-over effects, handling-induced stress, or cumulative physical fatigue cannot be entirely excluded. This dense testing schedule must be considered when interpreting the subtle variations in locomotor and cognitive outcomes.

Finally, despite its high biological activity, there are no data on the penetration of trans-vitisin B through the blood–brain barrier and its metabolism in vivo. A potential limitation of tVB as a central nervous system therapeutic candidate is its relatively high molecular weight (~906.9 g/mol), which theoretically restricts classical passive paracellular diffusion across the blood–brain barrier (BBB). Nevertheless, our in vivo findings demonstrate behavioral improvements and neuroprotective trends within the brain parenchyma. Chronic rotenone neurotoxicity likely causes this discrepancy by driving endothelial inflammation, which breaks down the BBB and allows larger molecules to enter the brain [46]. This inflammation weakens the barrier structure, directly increasing its permeability to molecules that would otherwise be excluded. These gaps highlight the clear need for further preclinical studies in more complex models and, should these prove successful, future evaluation in early-phase clinical trials in patients with early-stage PD to assess safety, bioavailability, and potential efficacy.

We believe that this study contributes to shifting the focus toward a better understanding of the potential conditions under which natural compounds exert their effects, representing an incremental step toward assessing the translatability of natural compounds into potential future therapeutic strategies.

The results of this study provide preclinical support for further investigation of alternative approaches to PD therapy. Current therapy focuses on dopamine replacement, a strategy that is effective in the early stages but does not halt disease progression and causes severe side effects. The data obtained with tVB suggest that a strategy aimed at preserving neurons and maintaining neural network function may offer a complementary approach, though extensive further research is required to determine its clinical relevance.

## 5. Conclusions

In the experimental models used, tVB demonstrates multi-level neuroprotection, including: (1) inhibition of neuroinflammation (reduction in IL-1β, TNF-α, COX-2, IBA-1); (2) suppression of oxidative stress (reduction in ROS, NO, nNOS, GSS); (3) attenuation of the loss of the dopaminergic neuronal population (TH+); and (4) improvement of behavioral functions. This makes it one of the few natural compounds shown in preclinical models to simultaneously affect key pathogenetic pathways of PD—from inflammation to proteopathy and oxidative damage. In this study, tVB exhibited a complex “systemic” neuroprotective effect across multiple readouts relevant to the pathogenesis of PD: from restoring proteostasis through the HSP70 chaperone network and suppressing p-Tau aggregation to maintaining microglia in an “anti-inflammatory” phenotype and preserving dopaminergic neurons without inducing the dyskinesia-like side effects observed with L-DOPA in this model. Unlike L-DOPA, which masks symptoms through excessive dopaminergic stimulation, the data suggest that tVB functions as an endoregulator of cellular stability, which was associated with improved motor and cognitive outcomes in this chronic rotenone model. These preclinical findings position tVB among the interesting candidates for further investigation in the context of PD with cognitive impairment (PDD) and support the rationale for additional preclinical toxicological and pharmacological studies to further characterize its profile prior to any consideration of clinical development.

Despite issues related to bioavailability and the lack of reliable clinical evidence confirming long-term safety and efficacy, oligomeric stilbenes remain attractive targets for preclinical research aimed at identifying agents that may slow the neurodegeneration observed in PD. Further research should focus on optimal dosing regimens, delivery methods, pharmacokinetics, biodistribution, and synergistic interactions between different classes of phytochemicals to better define their potential therapeutic outcomes while minimizing side effects.

## Data Availability

The original contributions presented in this study are included in the article/Supplementary Material. Further inquiries can be directed to the corresponding author(s).

## Supplementary Materials

The following supporting information can be downloaded at: https://www.mdpi.com/article/10.3390/antiox15091112/s1, Figure S1: HPLC profile of tVB, Figure S2: UV profile of tVB, Figures S3 and S4: Mass-spectra of tVB, Figures S5–S11: NMR-spectra of tVB.

## Abbreviations

The following abbreviations are used in this manuscript:

tVB	*trans*-vitisin B
PD	Parkinson’s disease
ROS	Reactive oxygen species
L-DOPA	l-3,4-dihydroxyphenylalanine
