# Supplementary material for: Trans-Vitisin B Targets Neuroinflammation, Oxidative Stress, and Tau Pathology to Improve Behavioral Outcomes in a Mouse Model of Parkinson’s Disease
Source: Antioxidants (Basel). 2026 Sep 3;15(9):1112. doi: 10.3390/antiox15091112 (PMC13603367; doi:10.3390/antiox15091112)
Supplement: Supplementary file 1 [file antioxidants-15-01112-s001.zip › antioxidants-4457504-supplementary.pdf]

Supplementary materials for manuscript

Trans-vitisin B targets neuroinflammation, oxidative stress, and tau pathology to improve behavioral outcomes in a mouse model of Parkinson's disease

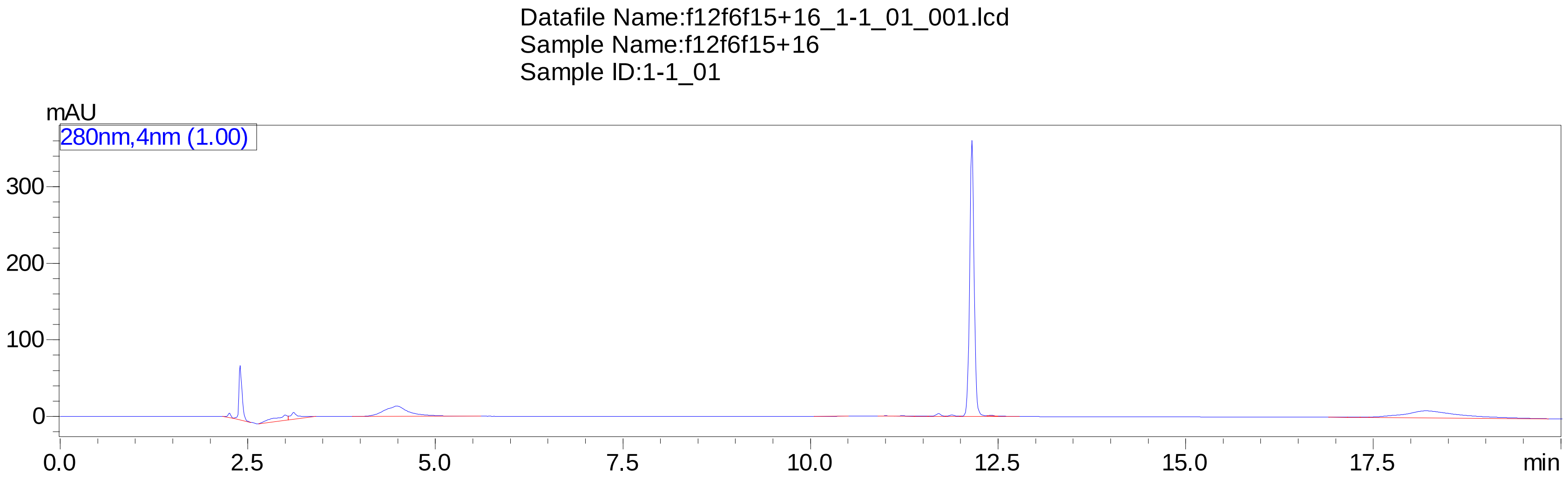

Figure S1. HPLC profile of tVB.

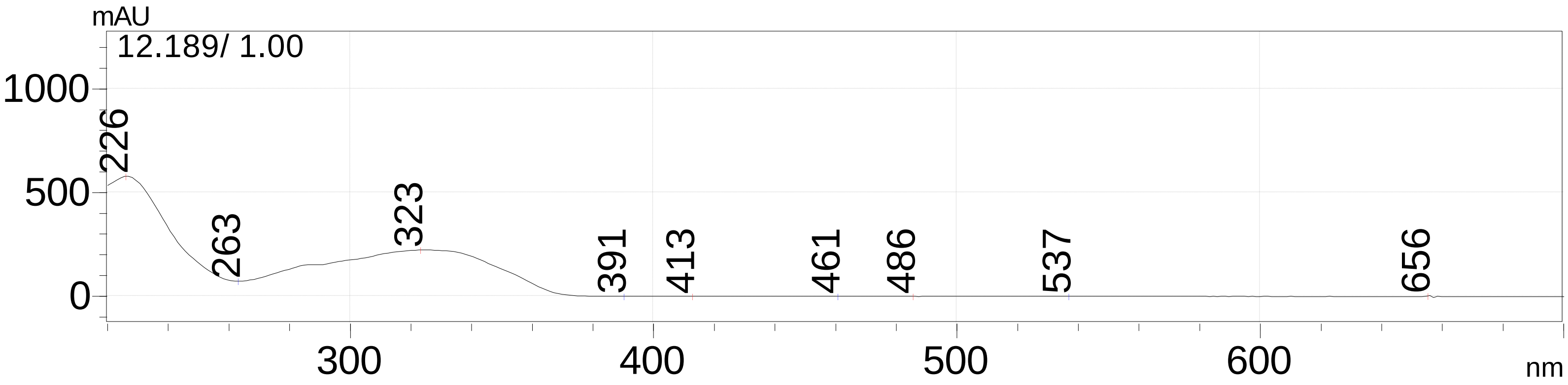

Figure S2. UV spectrum of tVB.

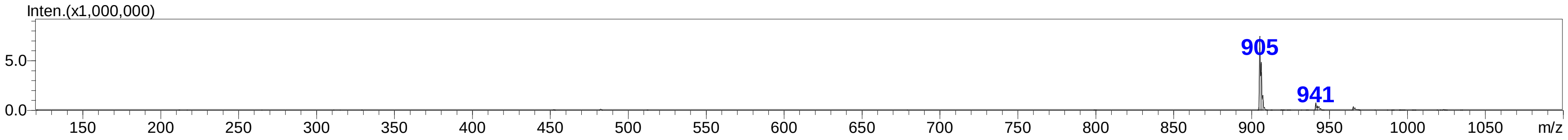

Figure S3. Mass-spectrum of tVB (positive ion mode).

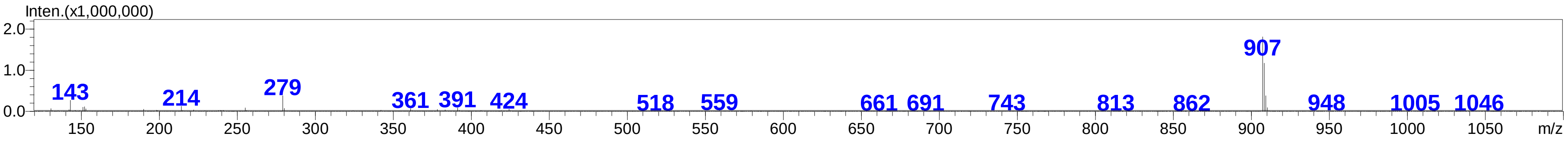

Figure S4. HR-MS spectrum of tVB (negative ion mode).

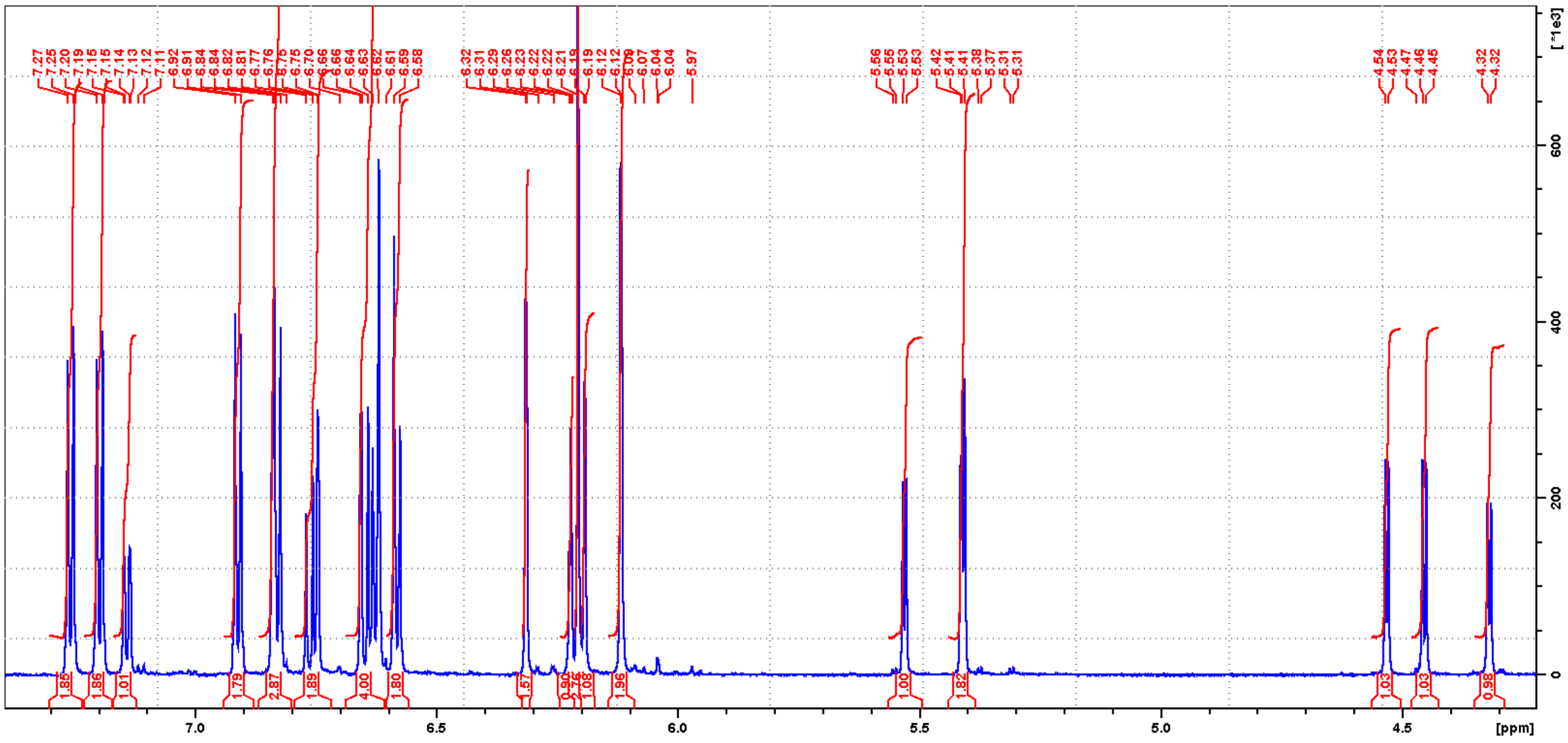

Figure S5. ^{1}H NMR spectrum of compounds tVB.

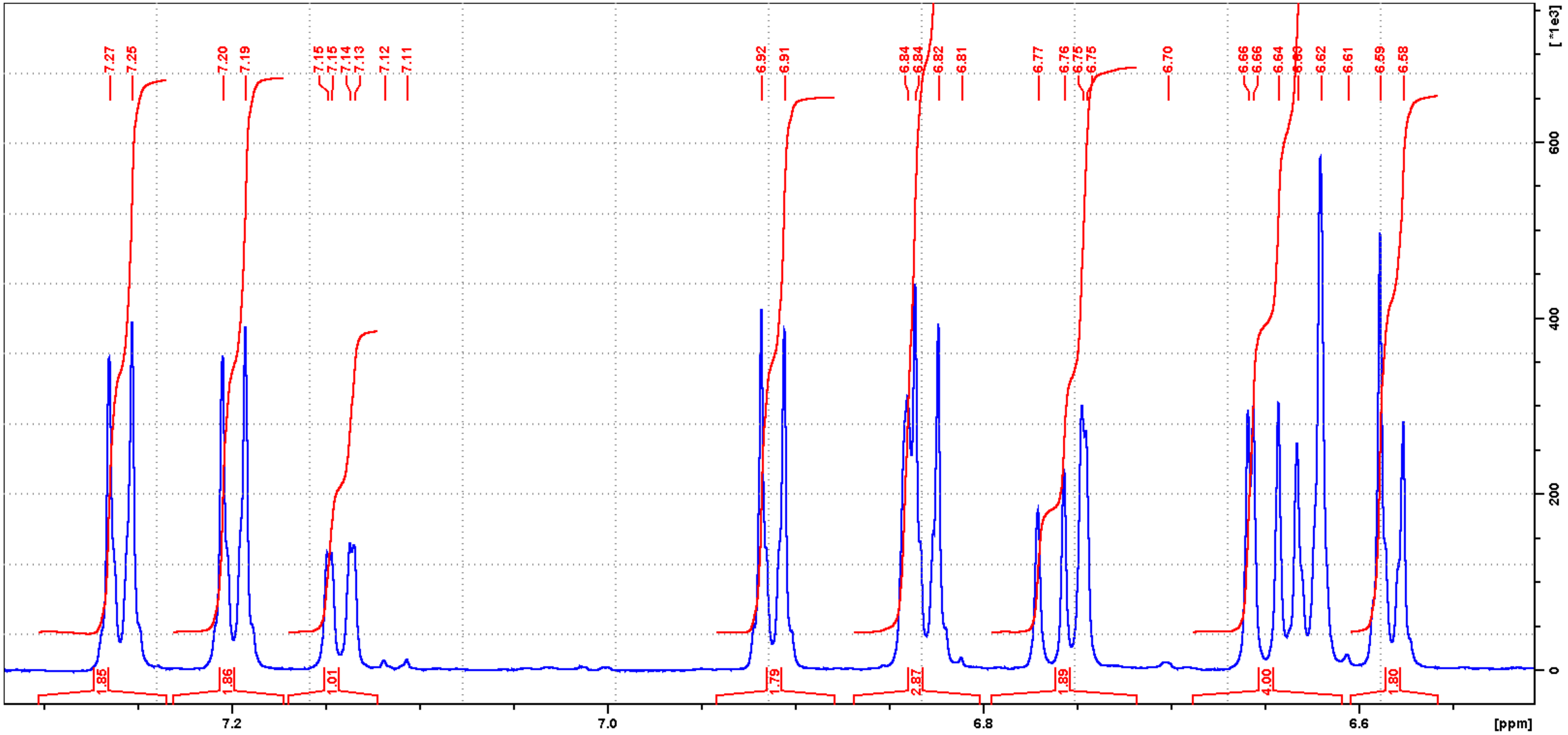

Figure S6. ^{1}H NMR spectrum of tVB (enlarged).

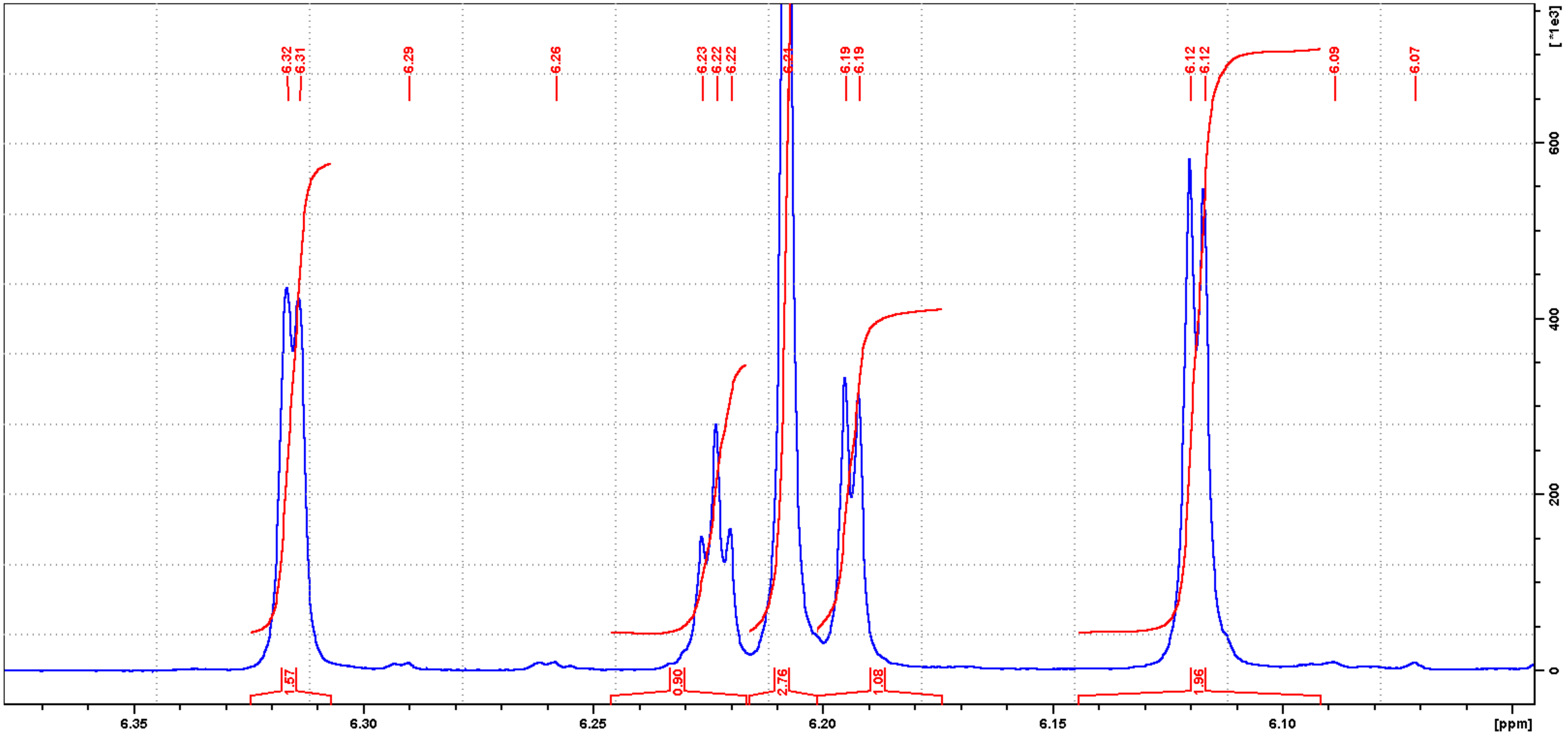

Figure S7. ^{1}H NMR spectrum of tVB (enlarged).

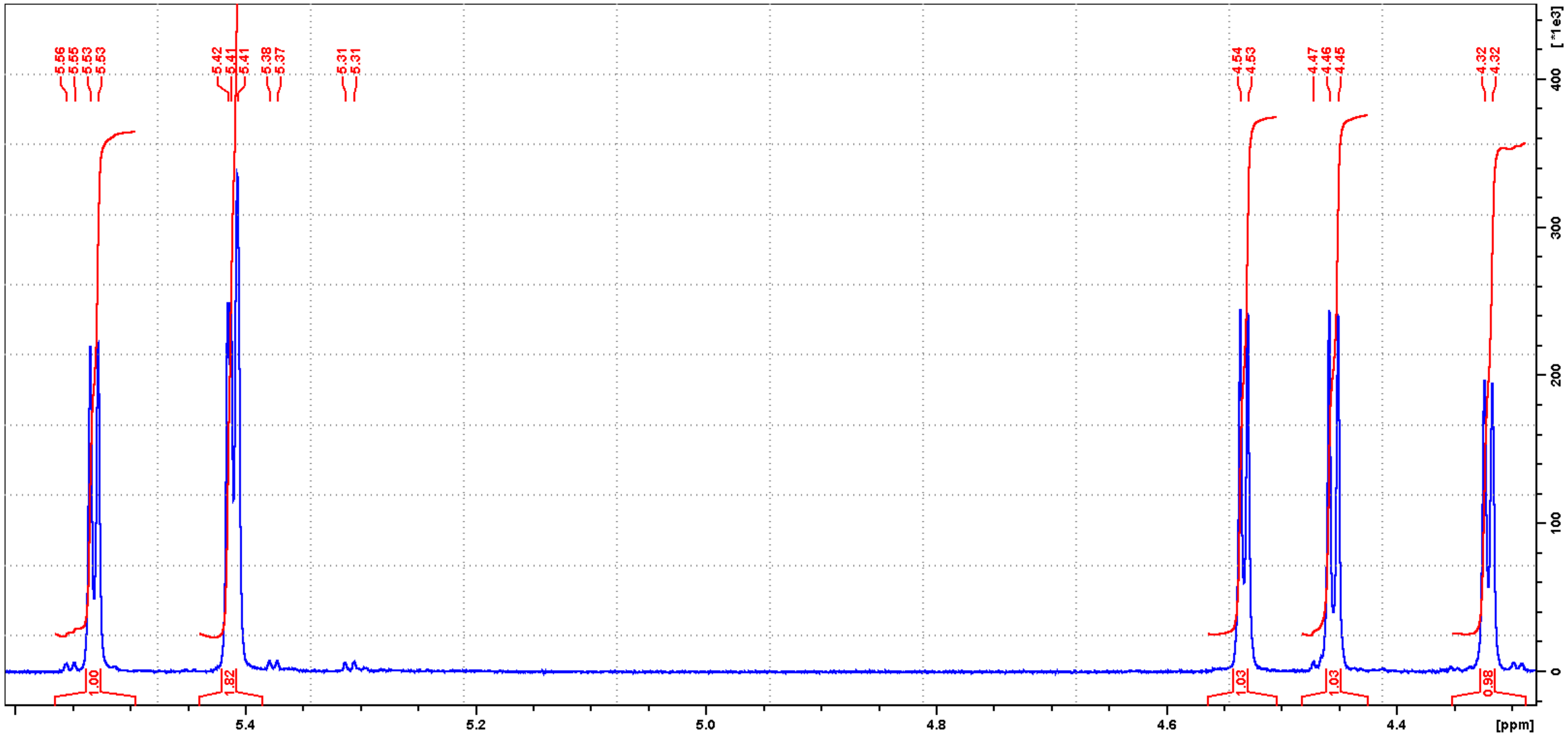

Figure S8. ¹H NMR spectrum of tVB (enlarged).

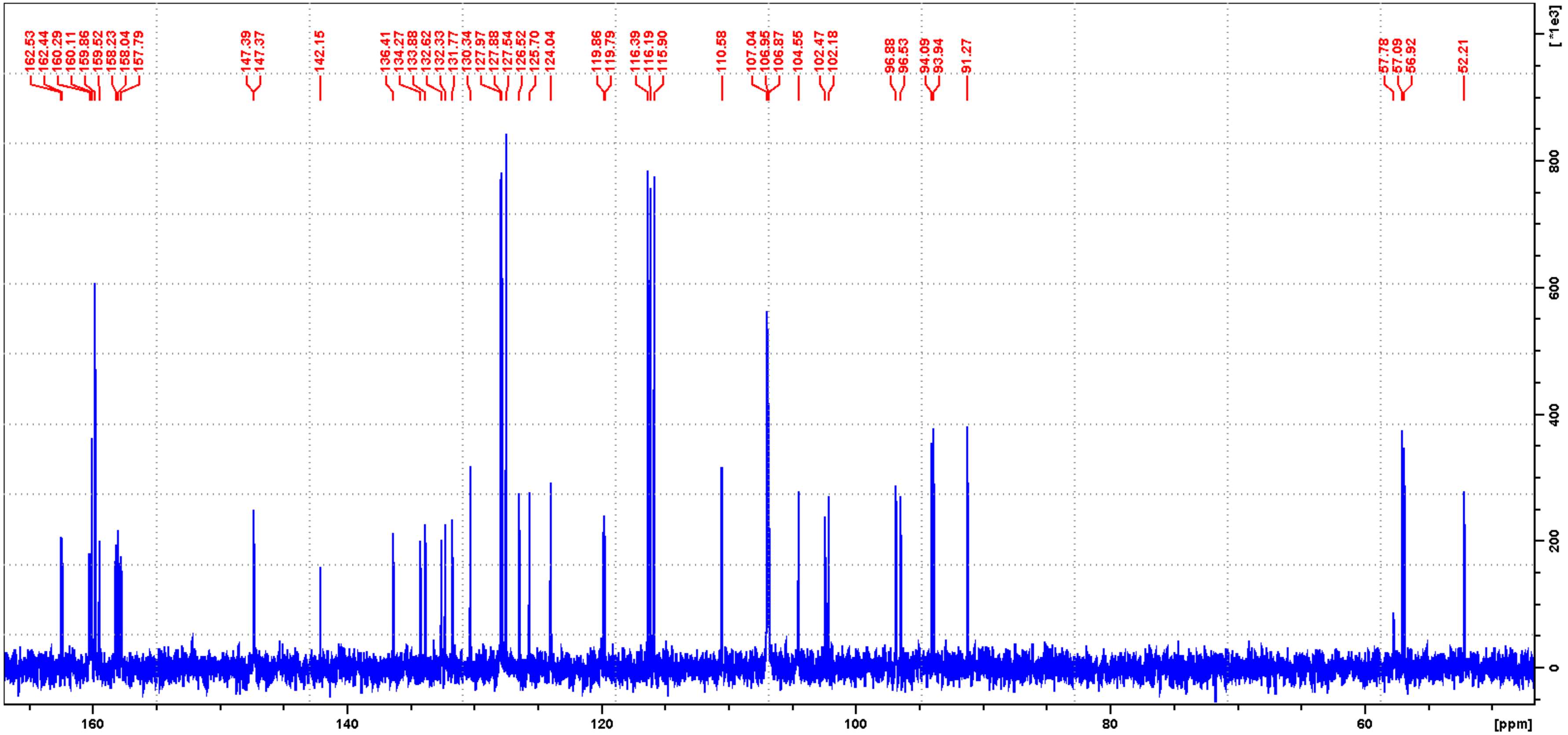

Figure S9. ^{13}C NMR spectrum of compounds tVB.

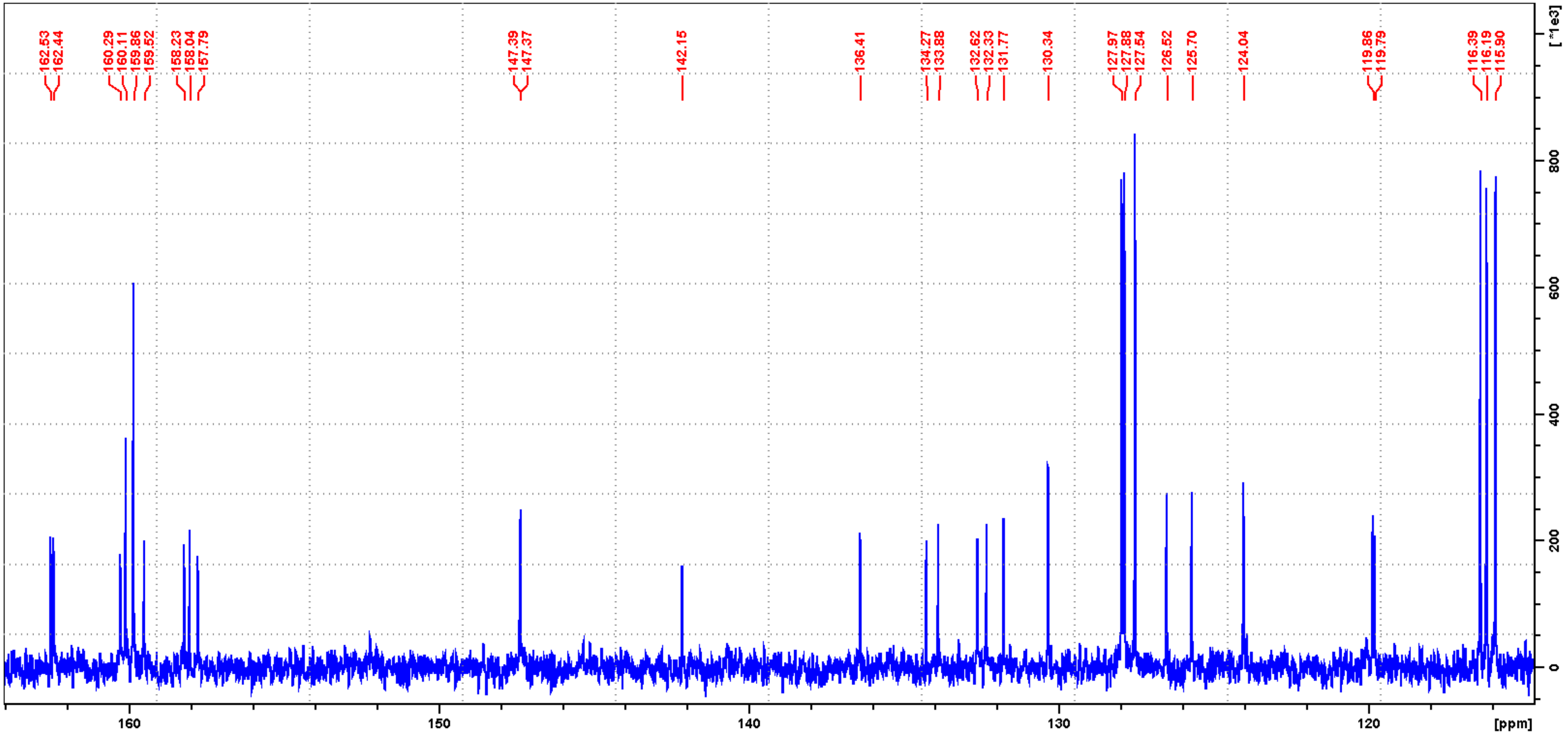

Figure S10. ^{13}C NMR spectrum of tVB (enlarged).

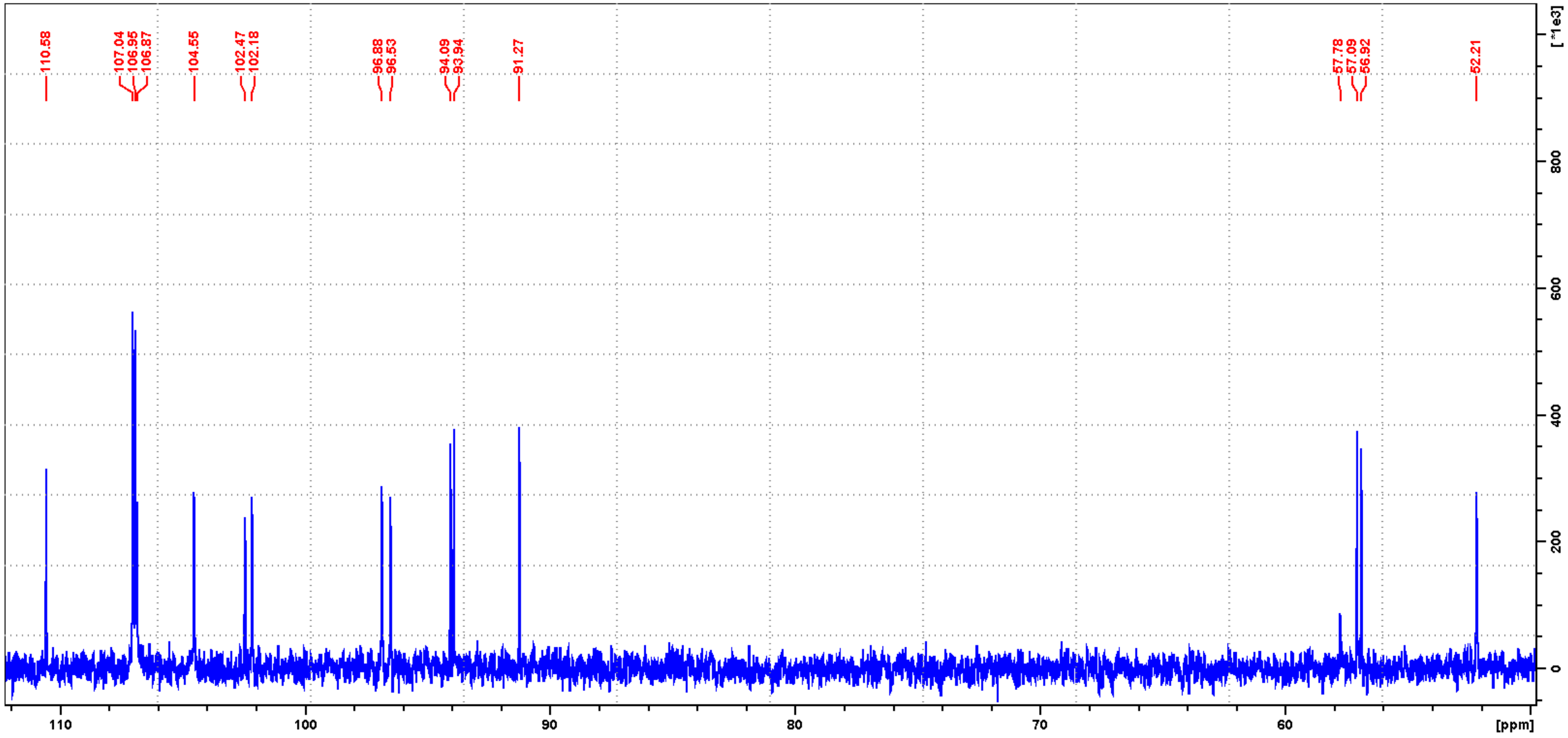

Figure S11. ^{13}C NMR spectrum of tVB (enlarged).
